# Efficiency of a Long-term Infectious Diseases Consultation and Antimicrobial Stewardship Program at a Japanese Cancer Center: An Interrupted Time-Series Analysis

**DOI:** 10.1093/ofid/ofae678

**Published:** 2024-11-13

**Authors:** Naoya Itoh, Nana Akazawa-Kai, Makoto Yamaguchi, Takanori Kawabata

**Affiliations:** Division of Infectious Diseases, Aichi Cancer Center Hospital, Aichi, Japan; Department of Infectious Diseases, Graduate School of Medical Sciences, Nagoya City University, Aichi, Japan; Department of Clinical Infectious Diseases, Graduate School of Medical Sciences, Nagoya City University, Aichi, Japan; Department of Infectious Diseases, Nagoya City University East Medical Center, Nagoya, Japan; Division of Infectious Diseases, Aichi Cancer Center Hospital, Aichi, Japan; Department of Infectious Diseases, Graduate School of Medical Sciences, Nagoya City University, Aichi, Japan; Department of Infectious Diseases, Nagoya City University East Medical Center, Nagoya, Japan; Division of Infectious Diseases, Aichi Cancer Center Hospital, Aichi, Japan; Department of Data Science, National Cerebral and Cardiovascular Center, Suita, Japan

**Keywords:** ABCDEFHIT criteria, antimicrobial stewardship program, cancer center, infectious diseases consultation, Japan

## Abstract

**Background:**

Patients with cancer are particularly susceptible to developing drug-resistant organisms due to the high frequency of infections during cancer treatment and the use of broad-spectrum antimicrobial agents. Therefore, patients with cancer are ideal candidates for an antimicrobial stewardship program (ASP); however, no established ASPs specifically target these patients. In this study, we evaluated the effect of a 46-month ASP intervention and infectious diseases consultation using a unique antimicrobial quality measure.

**Methods:**

Our single-center, retrospective, observational study was conducted from 1 April 2018 to 31 January 2024 and evaluated 2 phases: preintervention (antimicrobial notification by the infection control team) and postintervention (implementation of ASP and establishment of the infectious diseases consultation service).

**Results:**

The days of therapy (DOT) for 3 intravenous carbapenems significantly decreased, and the DOT of narrow-spectrum antimicrobials significantly increased after the intervention. A significant reduction was observed in the length of hospital stay, with no change in the incidence of hospital-acquired resistant microorganisms. All-cause in-hospital mortality rates and the 30-day mortality rate among patients with bacteremia episodes were numerically reduced, although not significantly, compared to the preintervention period. The rate of appropriate use of antimicrobial agents increased significantly during the late postintervention period (1 April 2021 to 31 January 2024).

**Conclusions:**

Our intervention was associated with the promotion of appropriate use of antimicrobial agents and a reduction in the length of hospital stay. These findings can help establish safer cancer treatments and improve patient prognosis.

## Teaser

A 46-month antimicrobial stewardship program for patients with cancer was associated with a significant reduction in carbapenem use and hospital stays, an improvement in the rate of antimicrobial use, and a numerical, though not significant, reduction in in-hospital and 30-day mortality rates among patients with bacteremia.

Cancer remains a leading cause of death worldwide, with approximately 10 million patients dying annually [1]. Although cancer-related mortality has continued to decline in recent years, infections remain a major cause of death, as patients are susceptible to serious infections while receiving chemotherapy, during surgery, and during the progression of the underlying disease [2–5]. Approximately 50% of patient deaths from cancer are infection-related or directly attributable to infections [6, 7]. For individuals with cancer, the risk of death from a fatal infection is approximately 3 times greater than that of individuals without cancer [7]. Additionally, infections in patients with cancer can delay scheduled chemotherapy or elective surgery, resulting in longer hospital stays, higher medical costs, and a negative impact on prognosis [8–10]. These patients are often overexposed to broad-spectrum antimicrobial agents such as carbapenems (CARs), which increases the risk of multidrug-resistant organisms [4]. In a study conducted at the National University Hospital in Singapore, Yeo et al reported that the use of CARs in patients with solid tumors and hematologic malignancies was approximately 4.5 times higher than that in other wards [11]. Furthermore, surgery, chemotherapy, prior hospitalization, a history of antimicrobial use, and severe neutropenia are associated with an increased risk of drug resistance in patients with cancer [12]. Antimicrobial stewardship programs (ASPs) can improve patient outcomes, decrease the incidence of antimicrobial resistance, and reduce healthcare-related costs through antimicrobial optimization [13]. Therefore, oncology patients are among the ideal candidates for ASPs.

However, ASPs for patients with cancer face significant challenges, and no established ASP exists owing to the uncertainty of infection diagnosis, the prevalence of multidrug-resistant bacterial infections, and the complexity of care required for immunocompromised patients [14, 15]. Limited studies on ASPs describing prospective audits and feedback and/or preauthorized implementation exist [4], because oncology patients are often excluded from studies on ASPs [11, 16–19]. The prevalence of drug-resistant organisms varies significantly across different countries and regions, making it crucial to assess the effectiveness of ASPs in different settings [20]. The use of broad-spectrum antimicrobials does not always imply inappropriate use, but there are no standardized criteria to assess the quality of ASPs [21]. We previously demonstrated that a 12-month intervention implementing ASP and infectious diseases (ID) consultations at a Japanese cancer center, using a unique antimicrobial quality measure referred to as the ABCDEFHIT criteria (Table 1), decreased the use of certain resistant organisms and broad-spectrum antimicrobials without negatively impacting patient outcomes [22]. Furthermore, our consultations on ASP and IDs have been shown to enhance the diagnostic process, further demonstrating the robustness of this intervention [23]. However, the effects of long-term ASP interventions are unknown, and validating their duration and effectiveness would provide useful information for facilities starting new ASPs. Therefore, the purpose of this study was to evaluate the effects of ASP and ID consultations over a 46-month period at a Japanese cancer center and to establish an ASP framework for the field of oncology.

**Table 1.** Assessment Sheet Used by the Antimicrobial Stewardship Team

Therapy	Category		
Appropriate therapy	A	Appropriate	Antimicrobial selection and dosage are appropriate at the time of evaluation.
	B	Better choice	There are no major problems with antimicrobial selection, although there are suggestions for some modifications and changes.
Inappropriate therapy	C	Culture	Absence or inadequacy of submission of bacterial cultures; requires additional investigation (or, therefore, is difficult to evaluate).
	D	De-escalation	Broad-spectrum antimicrobials were used based on the clinical characteristics, culture results, and local factors, and these can be changed to a narrow-spectrum antimicrobial.
	E	Escalation	The antimicrobials do not provide adequate coverage of the target microorganisms; therefore, the spectrum needs to be broadened or the antimicrobial should be changed.
	F	Fitting dose	The dose and method of administering the antimicrobials are inappropriate based on renal function or other factors; thus, adjustments are necessary.
	H	Halt	Discontinuation is necessary because the purpose of antimicrobial administration has been achieved, further use is unnecessary, or there is a risk of allergy.
	I	Indication document	The purpose of use and the target microorganisms of the antimicrobials are not described in the medical record and, therefore, cannot be evaluated; additional descriptions are needed.
Time out	T	Time out	Notify physician that culture results are available (3–5 days after initiation of antimicrobial therapy) or that it is time to reconsider the termination of antimicrobial therapy (10–14 days after initiation of antimicrobial therapy).

## METHODS

### Study Design and Setting

This study was conducted at the Aichi Cancer Center (ACC), a 500-bed tertiary care facility in Aichi, Japan. This single-institution retrospective observational study was conducted over 70 months between 1 April 2018 and 31 January 2024. Data for this study were obtained from the ACC database through microbiology profiles from the microbiology laboratory, prescription data from the pharmacy department, patient data from antimicrobial stewardship team (AST) conferences, and medical records. The AST in charge of the ASP comprised 1 ID physician (increased to 2 on 1 April 2021, and then to 3 after 1 April 2023), a pharmacist (not trained in ID), 2 laboratory technicians, and an infection control nurse (Supplementary Methods).

### Interventions

1. Preintervention period (1 April 2018 to 31 March 2020): notification system for specific antimicrobials to the infection control team. Before establishing the ID consultation service and ASP, the infection control team implemented a notification system for specific antimicrobial use without intervention, confirming the reason for use and the duration of treatment. Specific antimicrobials requiring notification included intravenous CARs (imipenem-cilastatin, meropenem, doripenem) and intravenous antimicrobials for anti–methicillin-resistant *Staphylococcus aureus* (MRSA): vancomycin, teicoplanin, daptomycin, and linezolid. Prescription decisions were determined by the primary physician team, with no intervention by an ID physician. Additionally, the management of *S aureus* bacteremia and candidemia was handled solely by the primary physician team.
2. Postintervention period (1 April 2020 to 31 January 2024): implementation of ASP and establishment of ID consultation service.

#### Antimicrobial Stewardship Program

On weekdays, ID specialists promptly communicated positive blood culture results to the primary physician team to ensure appropriate empirical therapy. However, over the weekend, neither the laboratory nor the ID specialists communicated blood culture results to the primary physician team; these were reported the following weekday instead. In cases where *S aureus* or *Candida* spp were detected, an ID consultation was recommended to improve prognosis, and the patients were examined at the bedside.

Audits were conducted 3 times weekly during AST conferences for all patients treated with specific intravenous anti-MRSA agents and certain broad-spectrum antibiotics (CARs, cefepime, cefozopran, and piperacillin-tazobactam). Beginning in April 2021, the audits were extended to include all weekdays, and intravenous fluoroquinolones (ciprofloxacin, levofloxacin) were added to the targeted antimicrobial list. In April 2022, CAR use required prior authorization and consultation with an ID specialist. When prescribing CAR after work hours and on holidays, the primary physician team contacted the ID physician on the following weekday. Patients who had already received an ID consultation were excluded from these audits. All cases reviewed at the conferences were documented in the electronic medical record system. AST members evaluated the intervention using the ABCDEFHIT criteria (Table 1) to qualitatively assess the appropriateness of specific antimicrobial use for the target cases based on culture results or treatment course and duration [22]. Evaluations were performed within 48 hours of initial treatment or during a period indicated by the AST (de-escalation or discontinuation when culture results or the AST suggested that imaging studies were required, and adjustment of antimicrobial doses when laboratory results were available). Evaluation decisions were made based on consensus among all AST members, and the results were classified. For patients deemed by the AST to require either modification of antimicrobial therapy or additional culture tests because of inadequate assessment, feedback was provided to the primary physician team on the audit day, either telephonically or via a note in the patients’ medical records. All audited patients were monitored daily on weekdays until the completion of their specific antimicrobial therapy or for the duration required by the AST. The AST contacted the primary physician teams whenever necessary. Any disagreement between the AST recommendations and the primary physician team's views was resolved through discussion, and the final decision was based on the physician team's judgment. In cases where bedside evaluation by ID specialists was required owing to challenges in diagnosing and determining the treatment for IDs based on medical record reviews at AST conferences, the ID specialists contacted the primary physician team to propose a consultation (Supplementary Methods).

#### ID Consultation Service

A dedicated ID specialist consulted with the primary physician team referred by the 15 aforementioned departments (Supplementary Methods) on all weekdays.

### Outcome Measures

The primary outcome measure was the change in days of therapy (DOT) with intravenous CAR (CAR-DOT; for imipenem–cilastatin, meropenem, and doripenem), expressed as DOT per 100 patient-days per month. This study focused on CAR, due to its broad spectrum, making its appropriate use particularly important.

The secondary outcome measures included the DOT for 3 antipseudomonal agents (piperacillin-tazobactam, cefepime, and cefozopran), 2 fluoroquinolones (ciprofloxacin and levofloxacin), 4 narrow-spectrum antimicrobials (ampicillin, ampicillin-sulbactam, cefazolin, and cefmetazole), 4 anti-MRSA antimicrobials (vancomycin, teicoplanin, daptomycin, and linezolid), all antimicrobials targeted for intervention, oral fluoroquinolones (ciprofloxacin, levofloxacin, and moxifloxacin), all intravenous antimicrobials, all oral antimicrobials, and the total of both intravenous and oral antimicrobials, along with antimicrobial use density (AUD) for these agents (Supplementary Methods). Other measures included the incidence of hospital-acquired resistant microorganisms, *Clostridioides difficile* infection (CDI), candidemia [24–27], the cost of CAR and all intravenous antimicrobials, the number of each type of culture sample per 1000 patient-days of hospitalization, 2-set blood culture rate, blood culture positivity rate, all-cause 30-day mortality rate of patients with blood culture–positive episodes, all-cause in-hospital mortality rate, length of hospital stay, and the assessment and acceptance rates of AST recommendations (Supplementary Methods).

### Statistical Analyses

From 1 April 2018 to 31 January 2024, an interrupted time-series analysis was conducted for the primary and secondary outcomes. To account for autocorrelation, which can lead to downward-biased standard errors and anti-conservative results when using the ordinary least squares estimator, we employed the Prais-Winsten estimator [28, 29]. Bivariate analysis was performed using the Mann-Whitney *U* test (continuous variables) with *P* < .05 considered statistically significant. Given the exploratory nature of the study, aimed at generating hypotheses, we did not apply multiplicity adjustments across the primary and secondary endpoints, recognizing the potential for increased type I errors. R software, version 4.2.0 (R Foundation for Statistical Computing, Vienna, Austria), was used for all statistical analyses.

### Ethical Considerations

The study protocol was approved by the Institutional Review Board of the ACC (approval number: 2023-0-252) and the study was conducted according to the principles of the Declaration of Helsinki. The requirement for informed consent was waived by the Institutional Review Board because this study only used data collected in clinical practice.

## RESULTS

### Cohort

During the study period, 58 846 patients were admitted to the ACC, with 24 461 (average of 894.2 ± 50.1 per month) in the preintervention period and 37 385 (average of 812.7 ± 97.2 per month) in the intervention period (*P* < .001). During the intervention period, the AST provided a total of 2599 feedback on specific antimicrobials. There were 3064 ID consultations throughout the intervention period.

### Antibiotic Consumption

#### Use of CARs

**Figure 1.**
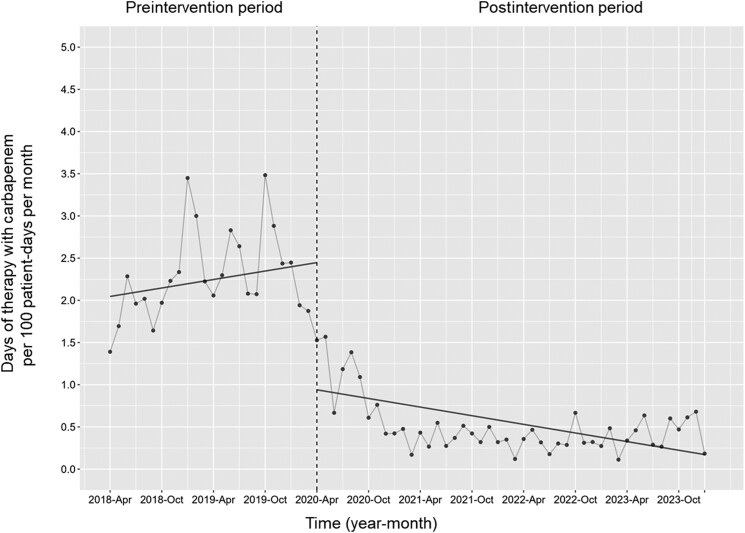
The days of therapy with carbapenem (CAR-DOT) per 100 patient-days per month. Each dot represents the CAR-DOT per 100 patient-days for each month. The slope is based on the linear regression analysis across 2 phases. The explanation of each phase is as follows: preintervention period (antimicrobial notification by the infection control team from 1 April 2018 to 31 March 2020); postintervention period (establishing an infectious diseases consultation service and implementation of the antimicrobial stewardship program from 1 April 2020 to 31 January 2024). The level of the monthly CAR-DOT significantly decreased (coefficient: −1.51 [95% confidence interval {CI}, −2.04 to −.97]; *P* < .001), although its trend did not decline (coefficient: −0.03 [95% CI, −.07 to .002]; *P* = .06).

Figure 1 shows the changes in CAR-DOT during the 2 phases. The level of monthly CAR-DOT significantly decreased (coefficient: −1.51 [95% confidence interval {CI}, −2.04 to −.97]; *P* < .001), although its trend did not decline (coefficient: −0.03 [95% CI, −.07 to .002]; *P* = .06).

#### Use of Antipseudomonal Agents

Supplementary Figure 1 shows the changes in the monthly DOT for the 3 antipseudomonal agents. No significant change was observed in the level or trend of the monthly DOT for the 3 antipseudomonal agents.

#### Use of Fluoroquinolones

Supplementary Figure 2 shows the changes in the monthly DOT for the 2 fluoroquinolones. No significant change was observed in the level or trend of the monthly DOT for the fluoroquinolones. Similarly, no significant change was observed in the level or trend of the monthly AUD for the fluoroquinolones.

#### Use of Narrow-Spectrum Antibiotics

**Figure 2.**
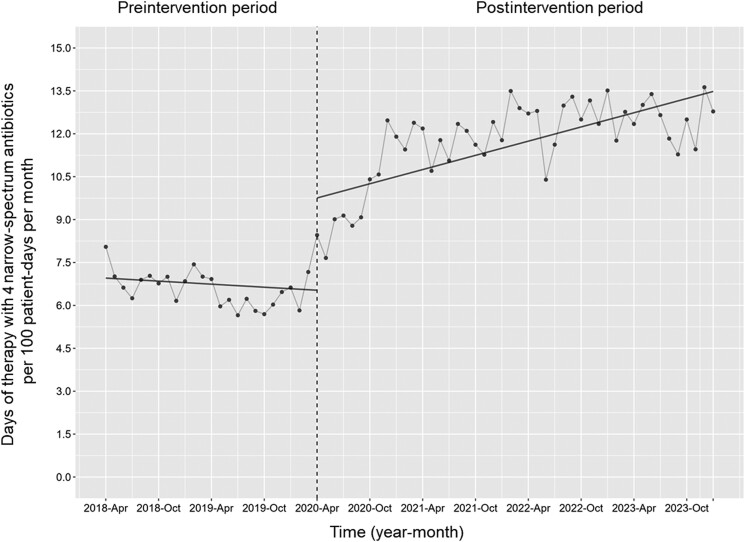
The days of therapy (DOT) with 4 narrow-spectrum antibiotics per 100 patient-days per month. Each dot represents the DOT with 4 narrow-spectrum antibiotics per 100 patient-days for each month. The slope is based on the linear regression analysis across 2 phases. The explanation of each phase is as follows: preintervention period (antimicrobial notification by the infection control team from 1 April 2018 to 31 March 2020); postintervention period (establishing an infectious disease consultation service and implementation of the antimicrobial stewardship program from 1 April 2020 to 31 January 2024). The level of the monthly DOT of the 4 narrow-spectrum antibiotics increased along with its trend (change in level, coefficient: 3.22 [95% confidence interval {CI}, 1.88–4.58], *P* < .001; trend change, coefficient: 0.10 [95% CI, .01–.20], *P* = .03).

Figure 2 shows the changes in the monthly DOT for the 4 narrow-spectrum antibiotics. The level in the monthly DOT of the 4 narrow-spectrum antibiotics increased along with its trend (change in level, coefficient: 3.22 [95% CI, 1.88–4.58], *P* < .001; trend change, coefficient: 0.10 [95% CI, .01–.20], *P* = .03).

#### Use of Anti-MRSA Antimicrobials

Supplementary Figure 3 shows the changes in the monthly DOT for the 4 anti-MRSA antimicrobials. No significant change was observed in the level or trend of the monthly DOT for the 4 anti-MRSA antimicrobials.

### Use of All Antimicrobials Targeted for Intervention

Supplementary Figure 4 shows the changes in the monthly DOT for all antimicrobials targeted for intervention. No significant change was observed in the level or trend of the monthly DOT for these antimicrobials.

### Use of Oral Fluoroquinolones

Supplementary Figure 5 shows the changes in the monthly DOT for oral fluoroquinolones. No significant change was observed in the level or trend of the monthly DOT for these antimicrobials.

### Use of All Intravenous Antimicrobials, All Oral Antimicrobials, and the Total of Both Intravenous and Oral Antimicrobials

Supplementary Figures 6, 7, and 8 show the changes in the monthly DOT for all intravenous antimicrobials, all oral antimicrobials, and the total of both intravenous and oral antimicrobials, respectively. No significant change was observed in the level or trend of the monthly DOT for all intravenous antimicrobials or the total of both intravenous and oral antimicrobials. The trend of monthly all oral antimicrobials significantly increased (coefficient: 0.15 [95% CI, .03–.27]; *P* = .02), although its level did not decrease.

### AUD for CARs, Antipseudomonal Agents, Narrow-Spectrum Antimicrobials, Fluoroquinolones, Anti-MRSA Antimicrobials, Oral Fluoroquinolones, All Intravenous Antimicrobials, All Oral Antimicrobials, and the Total of Both Intravenous and Oral Antimicrobials

The AUDs for these antimicrobials were consistent with their DOT results (Supplementary Results). The AUDs for these antimicrobials were consistent with their DOT results except for the significant increase in the level of all antimicrobials targeted for intervention (Supplementary Results).

### Incidence of Antimicrobial-Resistant Microorganisms, CDI, and Candidemia

During the study period, carbapenemase-producing Enterobacterales was not detected. No significant change was found in the level or trend of the monthly incidence of hospital-acquired resistant microorganisms, CDI, or candidemia (Supplementary Figures 9–14).

### Cost of CAR and All Intravenous Antimicrobials

Supplementary Table 1 shows the actual and adjusted monthly average CAR purchase costs for all intravenous antimicrobials during the study period. The actual CAR purchase cost per patient-day significantly decreased after the initiation of our intervention (*P* < .001). However, there was no significant change in the actual cost per patient-day for all intravenous antimicrobials purchased (*P* = .48). The adjusted CAR purchase, which considers the cost of switching from branded to generic products and changes in drug prices, significantly decreased the cost per patient-day after the initiation of our intervention (*P* < .001). Nonetheless, there was no significant change in the adjusted purchase cost per patient-day for all intravenous antimicrobials (*P* = .28).

### Culture Specimen Submissions

During the postintervention period, the level in the number of monthly total culture specimens (coefficient: 17.90 [95% CI, 11.6–24.2]; *P* < .001) and respiratory (coefficient: 4.44 [95% CI, 2.26–6.62]; *P* < .001), gastrointestinal (coefficient: 2.52 [95% CI, .69–3.66]; *P* < .001), urogenital (coefficient: 3.31 [95% CI, 2.18–4.45]; *P* < .001), and other specimens (coefficient: 3.70 [95% CI, .87–6.53]; *P* = .01) per 1000 patients increased. However, its trend did not change (Supplementary Figure 15). No significant changes were observed in the monthly levels of or trends in blood cultures or puncture fluid cultures per 1000 patients.

### Blood Culture Metrics

The 2-set rate of blood cultures was 84.3% ± 4.7% and 95.4% ± 2.4% before and after the intervention, respectively. The trend in the 2-set rate of monthly blood cultures did not increase after the intervention, but the level did significantly increase (coefficient: 6.5 [95% CI, 2.16–10.8]; *P* = .004) (Supplementary Figure 16). The rate of positive blood cultures was 10.2 ± 2.9 and 12.9 ± 3.0 before and after the intervention, respectively. No significant changes were found in the monthly levels or trends for the rate of positive blood cultures (Supplementary Figure 17).

### All-Cause In-Hospital Mortality and Length of Hospital Stay

**Figure 3.**
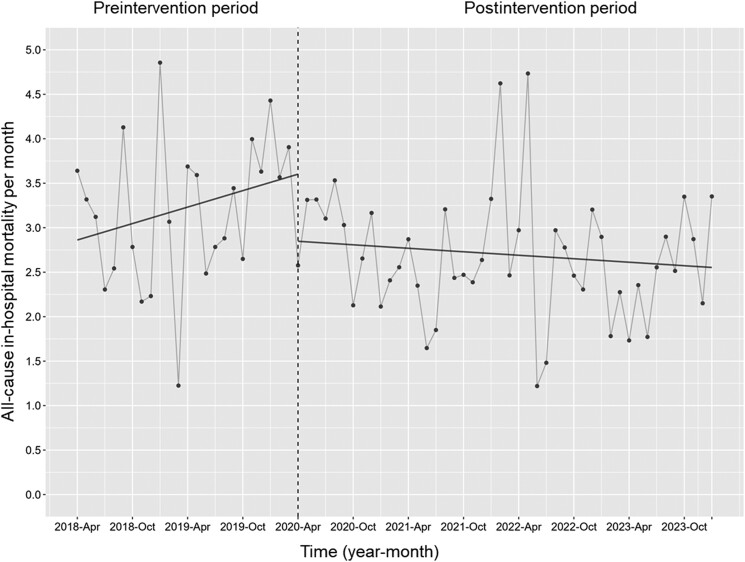
All-cause in-hospital mortality. Each dot on the graph signifies the all-cause in-hospital mortality rate for each month, with the slope calculated based on linear regression for the 2 phases. The explanation of each phase is as follows: preintervention period (antimicrobial notification by the infection control team from 1 April 2018 to 31 March 2020); postintervention period (establishing an infectious disease consultation service and implementation of the antimicrobial stewardship program from 1 April 2020 to 31 January 2024). No significant change was found in the level of the monthly all-cause in-hospital mortality rate (coefficient: −0.76 [95% confidence interval {CI}, −1.52 to .01]; *P* = .05) or its trend (coefficient: −0.04 [95% CI, −.08 to .01]; *P* = .12).

**Figure 4.**
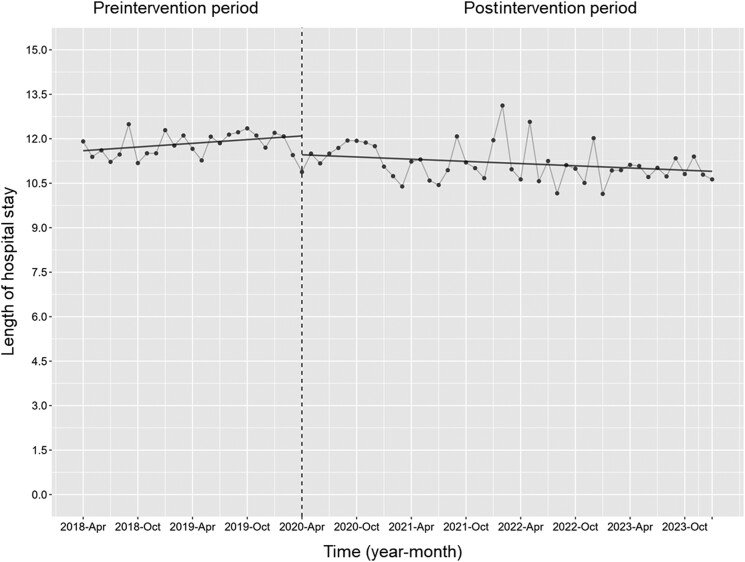
Length of hospital stay. Each dot on the graph signifies the duration of hospital stay each month, with the slope calculated based on linear regression for the 2 phases. The explanation of each phase is as follows: preintervention period (antimicrobial notification by the infection control team from 1 April 2018 to 31 March 2020); postintervention period (establishing an infectious disease consultation service and implementation of the antimicrobial stewardship program from 1 April 2020 to 31 January 2024). The level of the length of hospital stay decreased along with its trend (change in level, coefficient: −0.63 [95% CI, −1.17 to −.10], *P* = .02; trend change, coefficient: −0.03 [95% CI, −.07 to .0004], *P* = .047).

No significant changes were observed in the monthly levels or trends of the in-hospital mortality rate (Figure 3). However, the level in the length of hospital stay decreased along with its trend (change in level, coefficient: −0.63 [95% CI, −1.17 to −.10], *P* = .02; trend change, coefficient: −0.03 [95% CI, −.07 to −.0004], *P* = .047) (Figure 4).

### All-Cause 30-Day Mortality Rate of Patients With Blood Culture–Positive Episodes

A total of 1413 blood culture-positive episodes (preintervention, n = 434; postintervention, n = 1035) were included in the study. The mortality rate of bacteremia per episode was 17.4% ± 13.1% and 17.7% ± 8.54% pre- and postintervention, respectively. There was no significant change in the level of the monthly all-cause 30-day mortality rate of patients with blood culture–positive episodes (coefficient: −9.20 [95% CI, −18.4 to .01]; *P* = .05) or its trend (coefficient: −0.07 [95% CI, −.50 to .63]; *P* = .81) (Supplementary Figure 18).

### Assessment and Acceptance Rates of Proposals by the AST

Based on the evaluation by the AST, we determined that there were 1230 appropriate (47.3%) and 1369 inappropriate (52.7%) instances of antimicrobial use (Supplementary Table 2). The rate of appropriate evaluations was 38.8% ± 7.20% during the early intervention period (1 April 2020 to 31 March 2021) and 52.7% ± 9.01% during the late intervention period (1 April 2021 to 31 January 2024), showing a significant increase (*P* < .001). The overall acceptance rate of the AST suggestions was 81.2% ± 7.4%; the acceptance rate was 76.5% ± 5.48% during the early intervention period and 82.8% ± 7.41% during the late intervention period, showing a significant increase (*P* = .003).

## DISCUSSION

This is the first detailed study reporting the long-term effects of an ASP and ID consultation intervention at a cancer center in Japan. Based on its quality assessment criteria, the ASP was associated with a reduction in CAR use, without negative impact, and an improvement in the rate of appropriate antimicrobial use. All-cause in-hospital mortality and 30-day mortality rates among patients with bacteremia were reduced numerically, though not significantly, compared with the preintervention period.

CAR-DOT was reduced without increasing the use of alternative broad-spectrum antibiotics similar to carbapenems, indicating that our intervention did not merely substitute CAR with these alternatives. The lack of a significant reduction in antipseudomonal and fluoroquinolone antibiotic use was likely due to the cancer center routinely using these agents to treat febrile neutropenia caused by chemotherapy, which differs from general hospitals [30]. However, although the reduction displayed by these antimicrobials did not achieve statistical significance, a numerical reduction was observed and the clinical significance of our intervention is considered valid. The use of anti-MRSA agents did not change significantly; however, these agents may be used in both empirical and definitive therapy for catheter-related bloodstream infections in the cancer center [22, 23]. The acceptance rate of AST proposals and appropriate use rate significantly increased in the late intervention period, suggesting that specific antimicrobials were being used appropriately. Following the intervention, we observed an increase in the use of narrow-spectrum antibiotics, indicating effective de-escalation from empirical therapy. Additionally, except for the trend for all oral antimicrobials, there were almost no significant changes in the use of all antimicrobials targeted for intervention, oral fluoroquinolones, all intravenous antimicrobials, or the total of both intravenous and oral antimicrobials, suggesting that only the proportion of antimicrobials used changed as a result of our intervention. Although a causal relationship between the intervention and outcomes cannot be definitively established, the length of hospital stay was significantly reduced after the intervention. Moreover, while not statistically significant, a trend toward lower all-cause and 30-day mortality rates was observed in patients with bacteremia episodes. Our interventions are effective and novel, providing a strong rationale for actively implementing ASP and ID consultations in cancer centers.

Our intervention began in April 2020, but CAR usage had changed since early 2020. These changes might be attributed to a 1-hour lecture on appropriate antibiotic use given by an external ID physician to healthcare workers on 19 February 2020.

In our study, CAR use significantly decreased after the intervention. The use of broad-spectrum antimicrobials is not inherently inappropriate, as it varies among individual patients. Days of antibiotic spectrum coverage, a new indicator calculated by multiplying DOT by the antibiotic spectrum coverage score, encompasses the spectrum and quantity of antibiotics used [31]. Nevertheless, since the use of broad-spectrum antimicrobials is not always inappropriate, using this new indicator to qualitatively assess antimicrobial use remains challenging. Several studies have assessed antimicrobial use [32, 33] but have not established standardized quality criteria [21]. We used our ABCDEFHIT criteria to qualitatively assess specific antimicrobial use, showing a significant increase in appropriate use during the late intervention period and ensuring their efficient use in the hospital [22]. The ABCDEFHIT criteria, designed for AST conferences, are easily adaptable to other facilities. Therefore, the effectiveness of ASPs should be evaluated in the future using both quantitative measures of antimicrobials and standardized qualitative criteria.

Herein, the acceptance rate of AST suggestions significantly increased during the late intervention period. This increase could be attributed to ongoing telephonic communication of AST conference results to the primary physician team on a case-by-case basis, and to the establishment of trust through in-person ID consultations where the physician examined the patient. Notably, establishing a trusting relationship between the AST and the primary physician team is estimated to require at least 1 year.

We did not observe any changes in the incidence of drug-resistant bacteria. Although the association between antimicrobial resistance and the overuse of antimicrobials is well established, the effect of ASPs on reducing the incidence of drug-resistant bacteria varies across studies, and the results are inconsistent [19, 34, 35]. The effect of ASPs on drug-resistant bacteria in hospitalized patients may not be evident in short-term assessments, and our intervention requires longer-term observation [35].

Our intervention was associated with a significant reduction in the cost of purchasing CAR but did not reduce the overall cost of purchasing antimicrobials. However, it did significantly reduce the length of hospital stay, potentially leading to cost savings beyond material costs, suggesting the importance of evaluating other indicators besides antimicrobial costs when assessing the economic feasibility of ASPs [13].

In our study, the number of cultures for all specimens—respiratory, gastrointestinal, genitourinary, and other specimens—increased significantly after the intervention. The increase in the number of cultures submitted was favorable for making decisions on proposals, as AST conferences are mainly conducted at the desk. The significant increase in the rate of 2 sets of blood cultures was associated with more appropriate culture submissions. Although our study did not show a decrease in drug-resistant organisms, the increased number of specimen submissions might have contributed to the increased detection of potentially drug-resistant organisms.

This study had some limitations. First, it was conducted at a single cancer center in Japan, which limits the generalizability of the findings. However, based on the findings of both our previous and current studies, we believe that involving at least 1 ID specialist in ASPs can lead to meaningful results within at least 1 year, even in facilities with a high volume of broad-spectrum antimicrobial prescriptions [22]. Second, during the early stages of the coronavirus disease 2019 (COVID-19) pandemic, excessive antimicrobials were prescribed to patients with or suspected of having COVID-19 [36, 37], which should be considered. All patients with COVID-19 were managed by the ID physicians at our hospital, and unnecessary antimicrobial prescriptions were withheld. Furthermore, even during the COVID-19 pandemic, the use of CARs decreased, and no increase in the use of other broad-spectrum antimicrobial agents was reported. Finally, during the study period, our institution experienced several shortages of antimicrobials due to supply restrictions in Japan caused by manufacturing issues. Specifically, cefepime shortages occurred from June 2018 to October 2019, and cefazolin shortages from April to December 2019. In addition, imipenem-cilastatin shortages began in June 2021, while cefmetazole shortages started in June 2023 and continued until the end of the study period. The use of alternative antimicrobial agents increased substantially in some Japanese institutions owing to antimicrobial shortages, whereas the use of broad-spectrum antimicrobial agents decreased at our institution [38, 39]. This finding suggests that our intervention was appropriate and robust. To address these limitations, a long-term multicenter study involving other Japanese cancer centers is needed.

## CONCLUSIONS

This is the first study to evaluate the long-term effects of an ASP and ID consultations at a cancer center. These interventions were associated with improved antibiotic use, a reduction in the length of hospital stay, and a decrease in CAR use without a negative impact. These findings can facilitate the establishment of safer cancer treatment regimens and improve the prognosis for patients with cancer. Furthermore, this study highlights the critical role of ID specialists in cancer centers.

## Supplementary Data

Supplementary materials are available at *Open Forum Infectious Diseases* online. Consisting of data provided by the authors to benefit the reader, the posted materials are not copyedited and are the sole responsibility of the authors, so questions or comments should be addressed to the corresponding author.

## Supplementary Material

ofae678_Supplementary_Data

## Notes

***Acknowledgments*.** We are grateful to all the clinical staff of Aichi Cancer Center Hospital for their commitment toward providing patient care.

***Author contributions.*** N. I.: Conceptualization, data curation, funding acquisition, investigation, methodology, project administration, resources, validation, visualization, writing–original draft. T. K.: Formal analysis, methodology, software, validation, visualization. N. A.-K. and M. Y.: Validation. All authors reviewed the manuscript and approve its final, submitted version.

***Data availability.*** The datasets used and analyzed in the current study are available from the corresponding author upon reasonable request.

***Disclaimer.*** The content is solely the responsibility of the authors and does not represent the views of the sponsor.

***Financial support*.** This work was supported by the Japan Society for the Promotion of Science KAKENHI (grant numbers JP22K10547) and grants from the Department of Clinical Infectious Diseases, Graduate School of Medical Sciences, Nagoya City University.
