# Supplementary material for: Efficiency of a Long-term Infectious Diseases Consultation and Antimicrobial Stewardship Program at a Japanese Cancer Center: An Interrupted Time-Series Analysis
Source: Open Forum Infect Dis. 2024 Nov 13;11(12):ofae678. doi: 10.1093/ofid/ofae678 (PMC11597401; doi:10.1093/ofid/ofae678)
Supplement: ofae678_Supplementary_Data [file ofae678_supplementary_data.docx]

**Supplementary Appendix**

Supplement to: Efficiency of a Long-term Infectious Disease Consultation and Antimicrobial Stewardship Program at a Japanese Cancer Center: An Interrupted Time-Series Analysis

**Supplementary Methods**.........................................................................................................................4

Study design and setting................................................................................................................................4

Antimicrobial stewardship program..............................................................................................................4

Secondary outcome measures........................................................................................................................5

Days of therapy for antipseudomonal agents...............................................................................................5

Days of therapy for fluoroquinolones..........................................................................................................5

Days of therapy for narrow-spectrum antimicrobials..................................................................................6

Days of therapy for anti-methicillin-resistant *Staphylococcus aureu*s antimicrobials................................6

Days of therapy for all antimicrobials targeted for intervention.................................................................6

Days of therapy for oral fluoroquinolones...................................................................................................7

Days of therapy for all intravenous antimicrobials, all oral antimicrobials, and the total of both intravenous and oral antimicrobials.............................................................................................................7

Antimicrobial use density for CARs, antipseudomonal agents, narrow-spectrum antimicrobials, fluoroquinolones, anti-methicillin-resistant *S. aureus* antimicrobials, all antimicrobials targeted for intervention, oral fluoroquinolones, all intravenous antimicrobials, all oral antimicrobials, and the total of both intravenous and oral antimicrobials................................................................................................7

Incidence of hospital-acquired resistant microorganisms, *Clostridioides difficile* infection, and candidemia...................................................................................................................................................8

Cost of CAR and all intravenous antimicrobials.........................................................................................9

Number of each type of culture sample per 1,000 patient-days of hospitalization...................................10

Two-set blood culture rate.........................................................................................................................10

Blood culture positivity rate......................................................................................................................10

All-cause 30-day mortality rate of patients with blood culture-positive episodes.....................................11

All-cause in-hospital mortality rate and length of hospital stay................................................................11

Assessment and acceptance rate of AST recommendations......................................................................11

**Supplementary Results**..........................................................................................................................12

Cohort..........................................................................................................................................................12

The number of inpatients at the Department of Hematology and Cell Therapy........................................12

The average proportion of inpatients at the Department of Hematology and Cell Therapy......................12

The number of surgeries............................................................................................................................12

The average proportion of surgeries relative to the total number of inpatients.........................................13

Antimicrobial use density for CARs............................................................................................................13

Antimicrobial use density for antipseudomonal agents...............................................................................13

Antimicrobial use density for fluoroquinolones..........................................................................................13

Antimicrobial use density for narrow-spectrum antibiotics........................................................................14

Antimicrobial use density for anti-methicillin-resistant *S. aureus* antimicrobials......................................14

Antimicrobial use density for all antimicrobials targeted for intervention..................................................14

Antimicrobial use density for oral fluoroquinolones................................................ ..................................14

Antimicrobial use density for all intravenous antimicrobials, all oral antimicrobials, and the total of both intravenous and oral antimicrobials.............................................................................................................15

S1 Fig. The days of therapy with three antipseudomonal agents per 100 patient-days per month.............16

S2 Fig. The days of therapy with two fluoroquinolones per 100 patient-days per month...........................18

S3 Fig. The days of therapy with four anti-MRSA antimicrobials per 100 patient-days per month...........20

S4 Fig. The days of therapy with all antimicrobials targeted for intervention per 100 patient-days per month...........................................................................................................................................................22

S5 Fig. The days of therapy with oral fluoroquinolones per 100 patient-days per month..........................24

S6 Fig. The days of therapy with all intravenous antimicrobials per 100 patient-days per month.............26

S7 Fig. The days of therapy with all oral antimicrobials per 100 patient-days per month..........................28

S8 Fig. The days of therapy with the total of both intravenous and oral antimicrobials per 100 patient-days per month.............................................................................................................................................30

S9 Fig. The incidence of CRE per 1,000 patient-days per month...............................................................32

S10 Fig. The incidence of MRPA per 1,000 patient-days per month..........................................................34

S11 Fig. The incidence of MRSA per 1,000 patient-days per month..........................................................36

S12 Fig. The incidence of ESBL-producing Enterobacterales per 1,000 patient-days per month..............38

S13 Fig. The incidence of CDI per 1,000 patient-days per month..............................................................40

S14 Fig. The incidence of candidemia per 1,000 patient-days per month...................................................42

S15 Fig. The number of culture specimens per 1,000 patients per month..................................................43

S16 Fig. The two-set rate of blood cultures per month...............................................................................46

S17 Fig. The positive blood culture rate......................................................................................................47

S18 Fig. All-cause 30-day mortality rate of patients with blood culture-positive episodes........................48

S1 Table. Average monthly purchase cost per patient day for carbapenems and all intravenous antimicrobials from April 1, 2018, to January 31, 2024..............................................................................50

S2 Table. Content and acceptance rate of feedback by the AST with regard to specific antimicrobial usage............................................................................................................................................................51

**References**..................................................................................................................................................53

**Supplementary Methods**

**Study design and setting**

The center has 23 clinical departments and admits approximately 11,000 patients annually. Specifically, 15 departments are in charge of inpatients: Plastic and Reconstructive Surgery, Hematology and Cell Therapy, Thoracic Surgery, Thoracic Oncology, Gastroenterological Surgery, Gastroenterology, Orthopedic Surgery, Head and Neck Surgery, Breast Oncology, Neurosurgery, Urology, Gynecological Oncology, Radiation Oncology, Diagnostic and Interventional Radiology, and Clinical Oncology. The Department of Hematology and Cell Therapy treats patients with hematologic malignancies, while the other departments work with patients who have solid tumors. The Infection Control Team (ICT) consists of an infection control nurse, an infectious disease (ID) physician who also serves on the Antimicrobial Stewardship Team (AST) (increased to two on April 1, 2021, and then to three after April 1, 2023), two clinical laboratory technicians who are also AST members, and a respiratory medicine physician.

**Antimicrobial stewardship program**

For the AST conferences, the pharmacist retrieved patient data regarding specific antimicrobial treatments from the electronic medical record system of the Aichi Cancer Center (ACC). Laboratory technicians shared the latest microbiological information on these patients with other AST members. At the AST conference, both the pharmacist and the laboratory technicians offered their opinions on treatment management. When drug-resistant organisms were identified in a patient, the infection control nurse promptly alerted the manager of the ward to which the patient was admitted and provided necessary infection control measures. The ID physician oversaw the comprehensive management of each professional's role in this process.

As a general rule, culture results were not reported during off hours or on weekends, and were reported to the treatment team on the next weekday.

**Secondary outcome measures**

**Days of therapy for** **antipseudomonal agents**

The total days of therapy (DOT) per month, per 100 patient-days, was calculated for three intravenous antipseudomonal agents: piperacillin–tazobactam, cefepime, and cefozopran. This was because the aforementioned antibiotics are broad-spectrum antimicrobials similar to carbapenem (CAR).

**Days of therapy for fluoroquinolones**

The total DOT per month, per 100 patient-days, was calculated for two intravenous fluoroquinolones: ciprofloxacin and levofloxacin. The DOT for fluoroquinolones was evaluated to assess whether CAR was simply being replaced with other broad-spectrum agents.

**Days of therapy for narrow-spectrum antimicrobials**

Intravenous ampicillin, ampicillin/sulbactam, cefazolin, and cefmetazole were defined as narrow-range antimicrobial agents, and the total DOT per 100 patient-days per month was calculated for these four agents. These four antimicrobial agents were evaluated as reference indices for de-escalation from the broad-spectrum antimicrobial agents described above.

**Days of therapy for** **anti-methicillin-resistant *Staphylococcus aureus* antimicrobials**

Intravenous vancomycin, teicoplanin, daptomycin, and linezolid were included as anti-methicillin-resistant *S. aureus* (MRSA) antimicrobials, and the total DOT per 100 patient-days per month was calculated for these four agents.

**Days of therapy for all antimicrobials targeted for intervention**

Intravenous imipenem–cilastatin, meropenem, and doripenem, piperacillin–tazobactam, cefepime, cefozopran, ampicillin, ampicillin/sulbactam, cefazolin, cefmetazole, vancomycin, teicoplanin, daptomycin, and linezolid were included as all antimicrobials targeted for intervention, and the total DOT per 100 patient-days per month was calculated for these agents. These antimicrobial agents were evaluated as reference indices for assessing the overall dosage of our intervention agents.

**Days of therapy for** **oral fluoroquinolones**

The total DOT per month per 100 patient-days, was calculated for three oral fluoroquinolones: ciprofloxacin, levofloxacin, and moxifloxacin. The DOT for oral fluoroquinolones was evaluated to assess the oral switch from broad-spectrum antibacterial agents, including intravenous fluoroquinolones.

**Days of therapy for all intravenous antimicrobials, all oral antimicrobials, and the total of both intravenous and oral antimicrobials**

The total DOT per month, per 100 patient-days, was calculated for all intravenous antimicrobials, all oral antimicrobials, and all antimicrobials (including intravenous and oral). The DOT for these antimicrobial agents was evaluated to assess its use as a reference index to assess the impact of our intervention on the total volume of antimicrobial prescriptions for hospitalized patients.

**Antimicrobial use density for CARs, antipseudomonal agents, narrow-spectrum antimicrobials, fluoroquinolones, anti-methicillin-resistant *S. aureus* antimicrobials, all antimicrobials targeted for intervention, oral fluoroquinolones, all intravenous antimicrobials, all oral antimicrobials,** **and the total of both intravenous and oral antimicrobials**

To accurately and comprehensively determine the consumption of each antimicrobial agent, the antimicrobial use density (AUD) was also calculated for each antimicrobial. AUDs were calculated for three CARs (CAR-AUD; imipenem–cilastatin, meropenem, and doripenem), three antipseudomonal agents (piperacillin–tazobactam, cefepime, and cefozopran), four narrow-spectrum antimicrobials (ampicillin, ampicillin–sulbactam, cefazolin, and cefmetazole), two fluoroquinolones (ciprofloxacin and levofloxacin), anti-MRSA antimicrobials (vancomycin, teicoplanin, daptomycin, and linezolid), all these antimicrobials targeted for intervention, oral fluoroquinolones (ciprofloxacin, levofloxacin and moxifloxacin), all intravenous antimicrobials, all oral antimicrobials, and the total of both intravenous and oral antimicrobials.

**Incidence of hospital-acquired resistant microorganisms, *Clostridioides difficile* infection, and candidemia**

As an indicator of the ASP outcome, we measured the annual incidence of resistant microorganisms, *Clostridioides difficile* infection (CDI), and candidemia per 1,000 patient-days. The resistant microorganisms included carbapenemase-producing Enterobacterales (CPE), carbapenem-resistant Enterobacterales (CRE), multidrug-resistant *Pseudomonas aeruginosa* (MRPA), MRSA, and extended-spectrum beta-lactamase (ESBL)-producing Enterobacterales. Hospital-acquired microorganisms were defined as those that were identified >72 h after admission [2]. To exclude duplicates, when the same resistant microorganism was detected more than once in the same patient, only the first specimen obtained each month was included in the analysis [2]. However, if a resistant microorganism was detected in blood samples and the same resistant bacteria had not been detected in a blood sample from the same patient within the previous 2 weeks, the infection was defined as a new event [2]. Only clinical specimens of resistant microorganisms were included, and specimens for surveillance culture and negative confirmation were excluded. CDI was defined as the number of patients with evidence of CD toxin expression (C. DIFF QUIK CHEK COMPLETE; Alere Medical Co., Tokyo, Japan). Moreover, ESBL-producing Enterobacterales were identified using the Cica β-test (Kanto Chemical Co., Tokyo, Japan) and disc diffusion method. CPEs were identified using the modified carbapenem inactivation method, which was performed according to Clinical and Laboratory Standards Institute (CLSI) M100-S27 [3]. CRE was defined as either resistant to meropenem (minimum inhibitory concentrations [MIC] ≥2 μg/mL) or resistant to both imipenem (MIC ≥2 μg/mL) and cefmetazole (MIC ≥64 μg/mL) [4]. MRPA was defined as *P. aeruginosa* that was resistant to CAR and either aminoglycoside or fluoroquinolone in CLSI standard antimicrobial susceptibility tests, regardless of whether carbapenemase was produced [5]. Furthermore, we included the incidence of candidemia, although the blood samples intended for confirming negative candidemia were excluded from this analysis.

**Cost of CAR and all intravenous antimicrobials**

To assess the economic impact of the Antimicrobial Stewardship Program (ASP), we determined the per-patient-day cost of purchasing antimicrobials. The adjusted purchase costs were calculated based on the actual purchase costs (considering the cost of switching from branded to generic products and changes in drug prices) and prices of branded agents in January 2024. The exchange rate was calculated at 149 yen to 1 US dollar as of February 2024.

**Number of each type of culture sample per 1,000 patient-days of hospitalization**

To assess the impact of ID consultation and the application of the ASP, we evaluated the number of inpatient culture samples. Culture specimens for inpatients included blood, respiratory, gastrointestinal, and genitourinary specimens, puncture fluid, “other materials,” and total specimens. Respiratory specimens included sputum, pharyngeal mucus, nasal mucus, oral mucus, lung tissue, and bronchial lavage fluid. Gastrointestinal specimens encompassed stool, bile, and pancreatic fluid. Genitourinary specimens consisted of urine and vaginal secretions. Puncture fluid specimens comprised pleural fluid, ascitic fluid, spinal fluid, joint fluid, and bone marrow fluid. “Other materials” included catheter tips and wound cultures, drain cultures, and cultures obtained from alternative sources. Culture specimens used for screening were excluded from the study.

**Two-set blood culture rate**

The two-set blood culture rate was calculated as follows: (number of two sets/total sets) × 100.

**Blood culture positivity rate**

The blood culture positivity rate was calculated as follows: (number of positive sets/total sets) × 100.

**All-cause 30-day mortality rate of patients with blood culture-positive episodes**

To assess the impact of ID consultation and the application of the ASP, we evaluated the all-cause 30-day mortality rate of patients with blood culture-positive episodes. Second and subsequent episodes of bacteremia in the same patient during the study period were included in the mortality rate. In addition, the mortality rate included all causes of death, including cancer and IDs. Positive blood culture episodes with an unknown outcome were excluded from the calculation of all-cause 30-day mortality rate.

**All-cause in-hospital mortality rate and length of hospital stay**

To assess the impact of ID consultation and application of the ASP, we evaluated the all-cause in-hospital 30-day mortality rate and length of hospital stay.

**Assessment and acceptance rate of AST recommendations**

The AST calculated the evaluation (**Table 1**) and acceptance rates (the sum of fully and partially accepted suggestions divided by the total number of suggestions). A and B evaluations were defined and evaluated as appropriate use. These measures for the early intervention period (April 1, 2020, to March 31, 2021) and the late intervention period (April 1, 2021, to January 31, 2024) were compared.

**Supplementary Results**

**Cohort**

**The number of inpatients at the Department of Hematology and Cell Therapy**

The number of inpatients in the Department of Hematology and Cell Therapy was 846 (average of 35.3 ± 5.7 per month) in the pre-intervention period and 1,494 (average of 32.4 ± 8.9 per month) in the post-intervention period (p = 0.05).

**The average proportion of inpatients at the Department of Hematology and Cell Therapy**

No significant difference was found in the average proportion of inpatients at the Department of Hematology and Cell Therapy relative to the total number of inpatients between the pre-intervention period (average of 3.94 ± 0.6% per month) and the post-intervention period (average of 4.00 ± 1.0% per month) (p = 0.68).

**The number of surgeries**

The number of surgeries was 6,731 (average of 280.5±22.4 per month) during the pre-intervention period and 12,552 (average of 272.9±36.6 per month) during the post-intervention period (p=0.68).

**The average proportion of surgeries relative to the total number of inpatients**

The average proportion of surgeries relative to the total number of inpatients significantly increased in the post-intervention period (pre-intervention period: average of 31.4±2.25% per month; post-intervention period: average of 33.6±2.9% per month) (p=0.001).

**Antimicrobial use density for CARs**

The level of the monthly CAR-AUD significantly decreased (coefficient: -0.90, 95% CI: -‍1.26 to -0.54, p < 0.001), although its trend did not show a reduction (coefficient: -0.02, 95% CI: -0.05 to 0.001, p = 0.06).

**Antimicrobial use density for antipseudomonal agents**

There was no significant change in the level of the monthly AUD for the three antipseudomonal agents or their trend.

**Antimicrobial use density for fluoroquinolones**

No significant change was observed in the level of the monthly AUD for fluoroquinolones or their trend.

**Antimicrobial use density for narrow-spectrum antibiotics**

The level of the monthly AUD of the four narrow-spectrum antibiotics significantly increased (coefficient: 2.93, 95% CI: 1.58 to 4.27, p < 0.001), although their trend did not increase.

**Antimicrobial use density for anti-methicillin-resistant *S. aureus* antimicrobials**

There was no significant change in the level of the monthly AUD for the four anti-MRSA antimicrobials or their trend.

**Antimicrobial use density for all antimicrobials targeted for intervention**

The level of monthly all antimicrobials targeted for intervention increased (coefficient: 1.59, 95% CI: 0.19 to 3.00, p = 0.03), although its trend did not change.

**Antimicrobial use density for oral fluoroquinolones**

There was no significant change in the level of the monthly AUD for the oral fluoroquinolones or their trend.

**Antimicrobial use density for all intravenous antimicrobials, all oral antimicrobials, and the total of both intravenous and oral antimicrobials**

No significant change was observed in the level or trend of the monthly AUD for all intravenous antimicrobials, the total of both intravenous and oral antimicrobials. The trend of monthly all oral antimicrobials significantly increased (coefficient: 0.11, 95% CI: 0.005 to 0.21, p = 0.04), although its level did not decrease.

**S1 Fig. The days of therapy with three antipseudomonal agents per 100 patient-days per month**


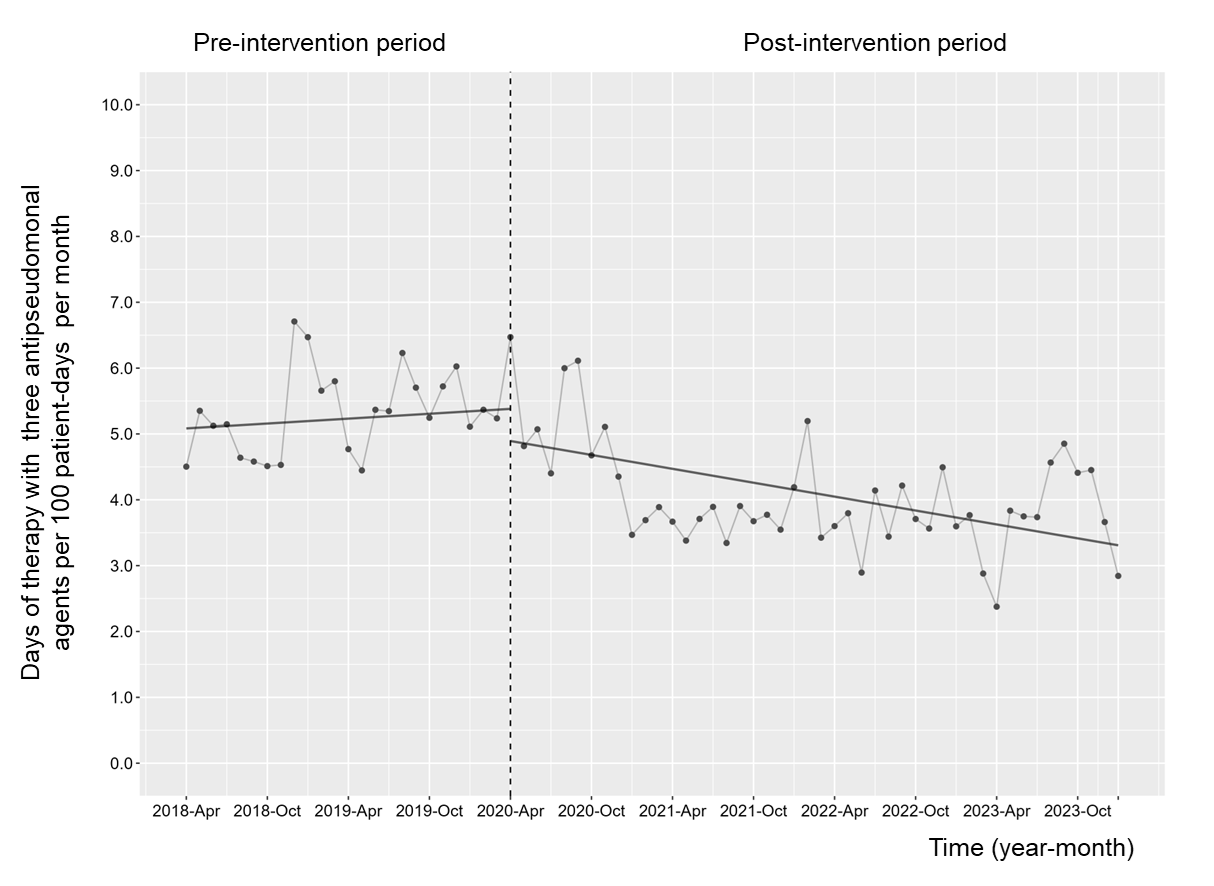


Each dot represents the days of therapy with three antipseudomonal agents per 100 patient-days for each month. The slope is based on the linear regression analysis across two phases. The explanation of each phase is as follows: **Pre-intervention period** (antimicrobial notification by the infection control team from April 1, 2018, to March 31, 2020); **Post-intervention period** (establishing an infectious disease consultation service and implementation of the Antimicrobial Stewardship Program from April 1, 2020, to January 31, 2024). No significant change was observed in the level of the monthly DOT for the three antipseudomonal agents (coefficient: -0.49; 95% CI: -1.48 to 0.50, p = 0.33) or its trend (coefficient: −0.05; 95% CI: -0.11 to 0.02, p = 0.15).

**S2 Fig. The days of therapy with two fluoroquinolones per 100 patient-days per month**


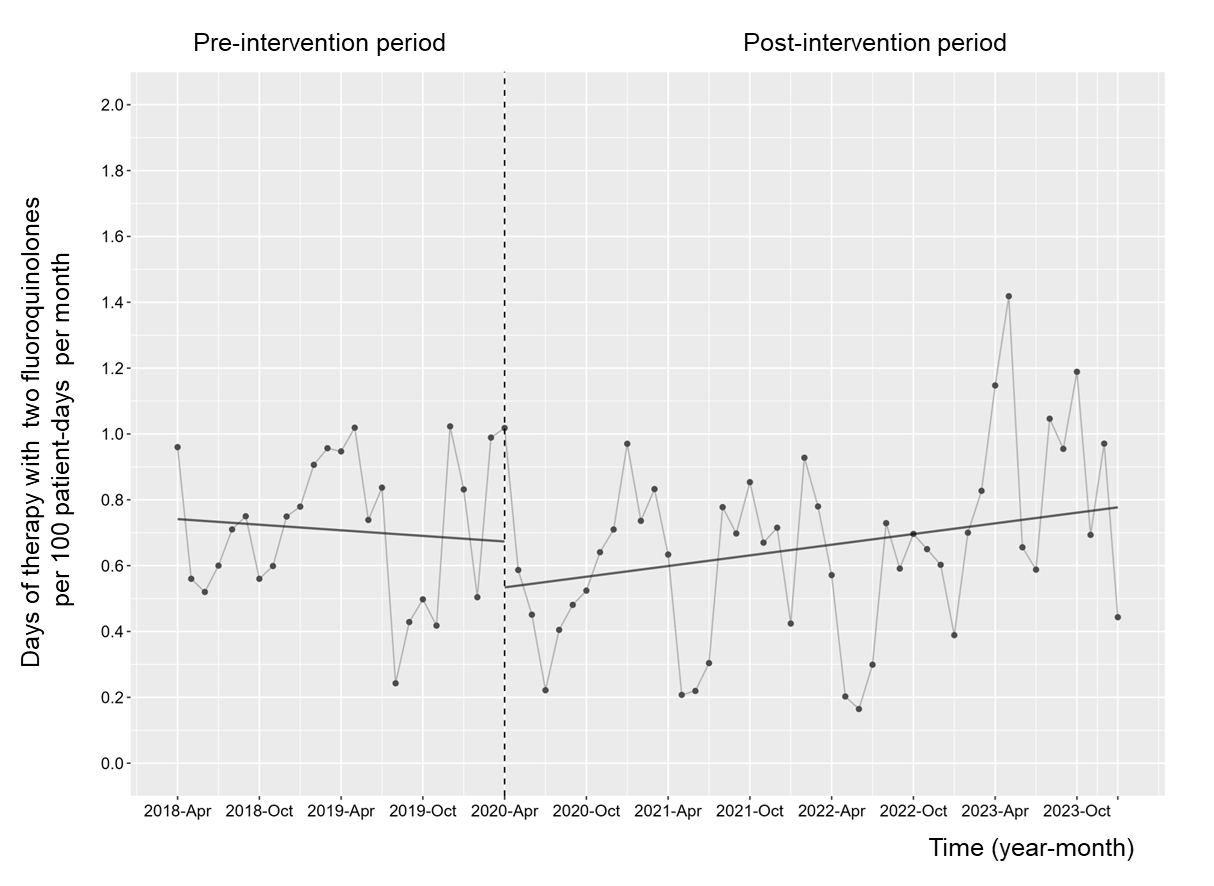


Each dot represents the days of therapy with two fluoroquinolones per 100 patient days for each month. The slope is based on the linear regression analysis across two phases. The explanation of each phase is as follows: **Pre-intervention period** (antimicrobial notification by the infection control team from April 1, 2018, to March 31, 2020); **Post-intervention period** (establishing an infectious disease consultation service and implementation of the Antimicrobial Stewardship Program from April 1, 2020, to January 31, 2024). No significant change was observed in the level of the monthly DOT for the fluoroquinolones (coefficient: -0.14; 95% CI: -0.49 to 0.21, p = 0.43) or their trend (coefficient: 0.01; 95% CI: -0.02 to 0.03, p = 0.48).

**S3 Fig. The days of therapy with four anti-MRSA antimicrobials per 100 patient-days per month**


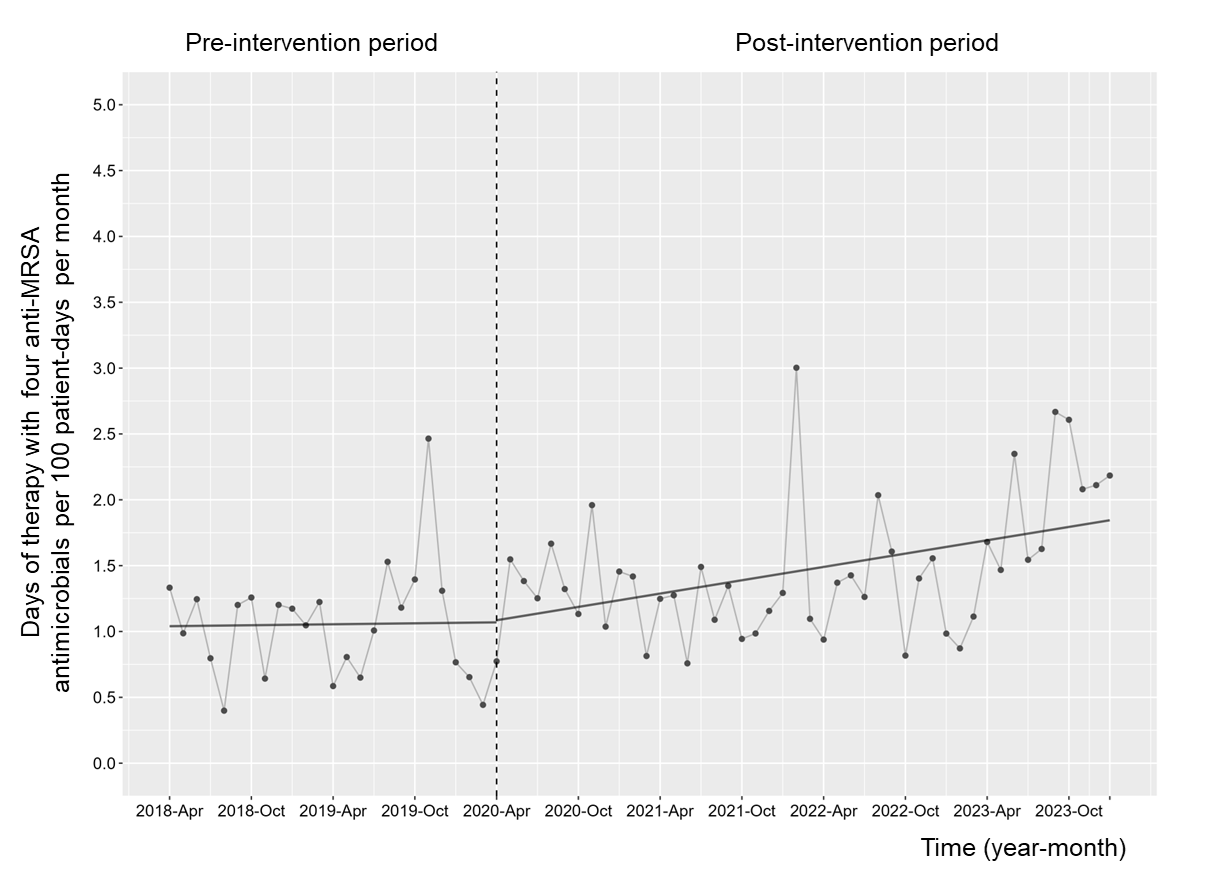


Each dot represents the days of therapy with four anti-methicillin-resistant *Staphylococcus aureus* (MRSA) antimicrobials per 100 patient-days for each month. The slope is based on the linear regression analysis across two phases. The explanation of each phase is as follows: **Pre-intervention period** (antimicrobial notification by the infection control team from April 1, 2018, to March 31, 2020); **Post-intervention period** (establishing an infectious disease consultation service and implementation of the Antimicrobial Stewardship Program from April 1, 2020, to January 31, 2024). No significant change was observed in the level of the monthly DOT for the four anti-MRSA antimicrobials (coefficient: 0.02; 95% CI: -0.54 to 0.58, p = 0.96) or their trend (coefficient: 0.02; 95% CI: -0.02 to 0.05, p = 0.38).

**S4 Fig. The days of therapy with all antimicrobials targeted for intervention per 100 patient-days per month**


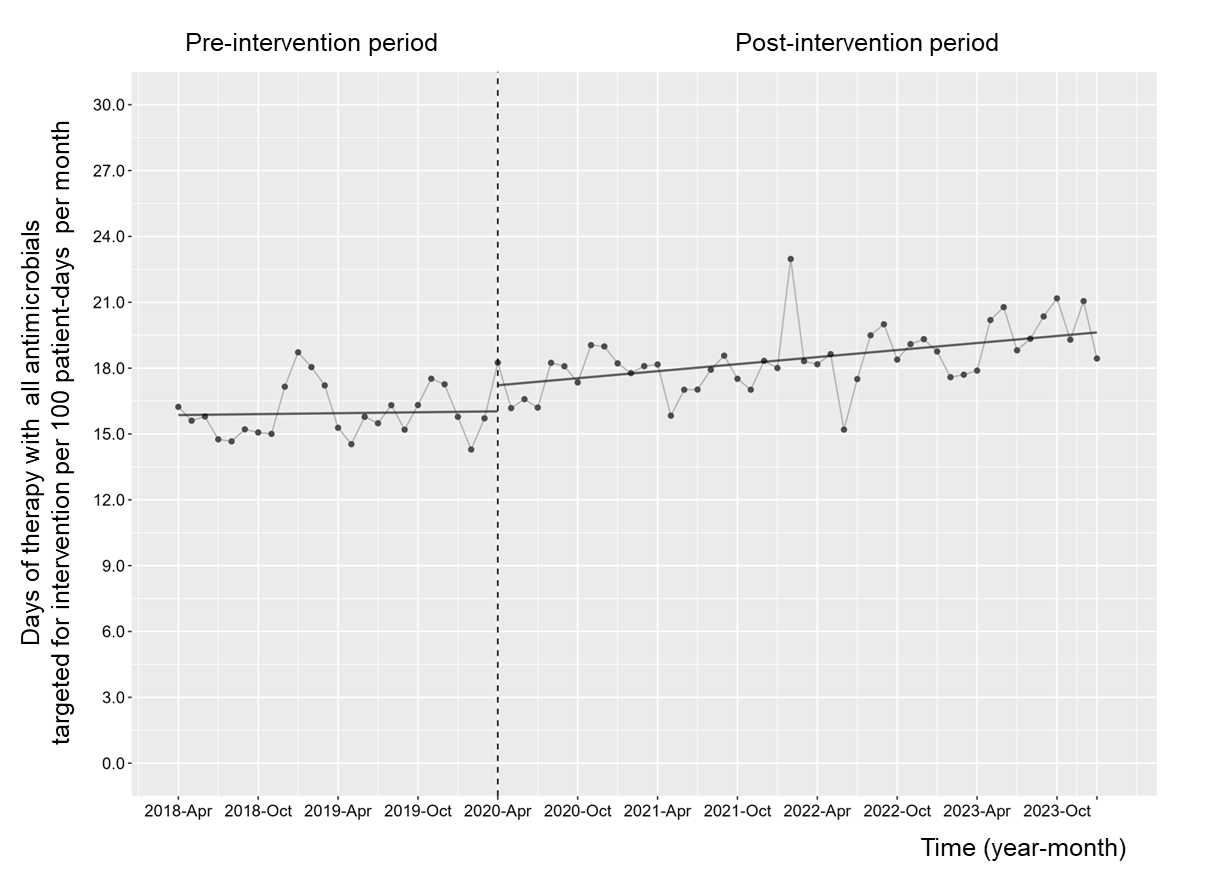


Each dot represents the days of therapy with all antimicrobials targeted for intervention per 100 patient-days for each month. The slope is based on the linear regression analysis across two phases. The explanation of each phase is as follows: **Pre-intervention period** (antimicrobial notification by the infection control team from April 1, 2018, to March 31, 2020); **Post-intervention period** (establishing an infectious disease consultation service and implementation of the Antimicrobial Stewardship Program from April 1, 2020, to January 31, 2024). No significant change was observed in the level of the monthly DOT for these antimicrobials (coefficient: 1.19; 95% CI: -0.37 to 2.75, p = 0.13) or their trend (coefficient: 0.05; 95% CI: -0.05 to 0.15, p = 0.35).

**S5 Fig. The days of therapy with oral fluoroquinolones per 100 patient-days per month**

**
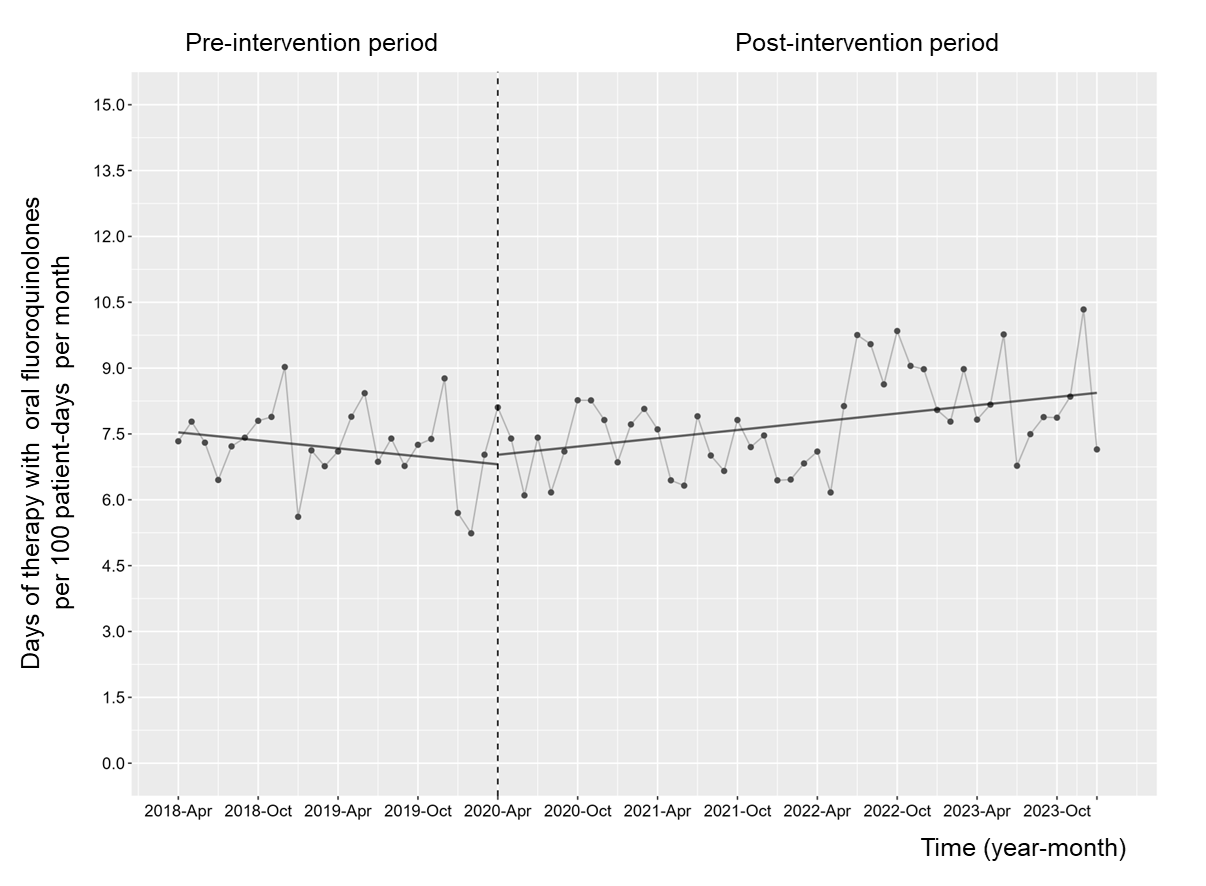
**

Each dot represents the days of therapy with oral fluoroquinolones per 100 patient-days for each month. The slope is based on the linear regression analysis across two phases. The explanation of each phase is as follows: **Pre-intervention period** (antimicrobial notification by the infection control team from April 1, 2018, to March 31, 2020); **Post-intervention period** (establishing an infectious disease consultation service and implementation of the Antimicrobial Stewardship Program from April 1, 2020, to January 31, 2024). No significant change was observed in the level of the monthly DOT for oral fluoroquinolones (coefficient: 0.22; 95% CI: -0.91 to 1.35, p = 0.70) or their trend (coefficient: 0.06; 95% CI: -0.009 to 0.13, p = 0.09).

**S6 Fig. The days of therapy with all intravenous antimicrobials per 100 patient-days per month**

**
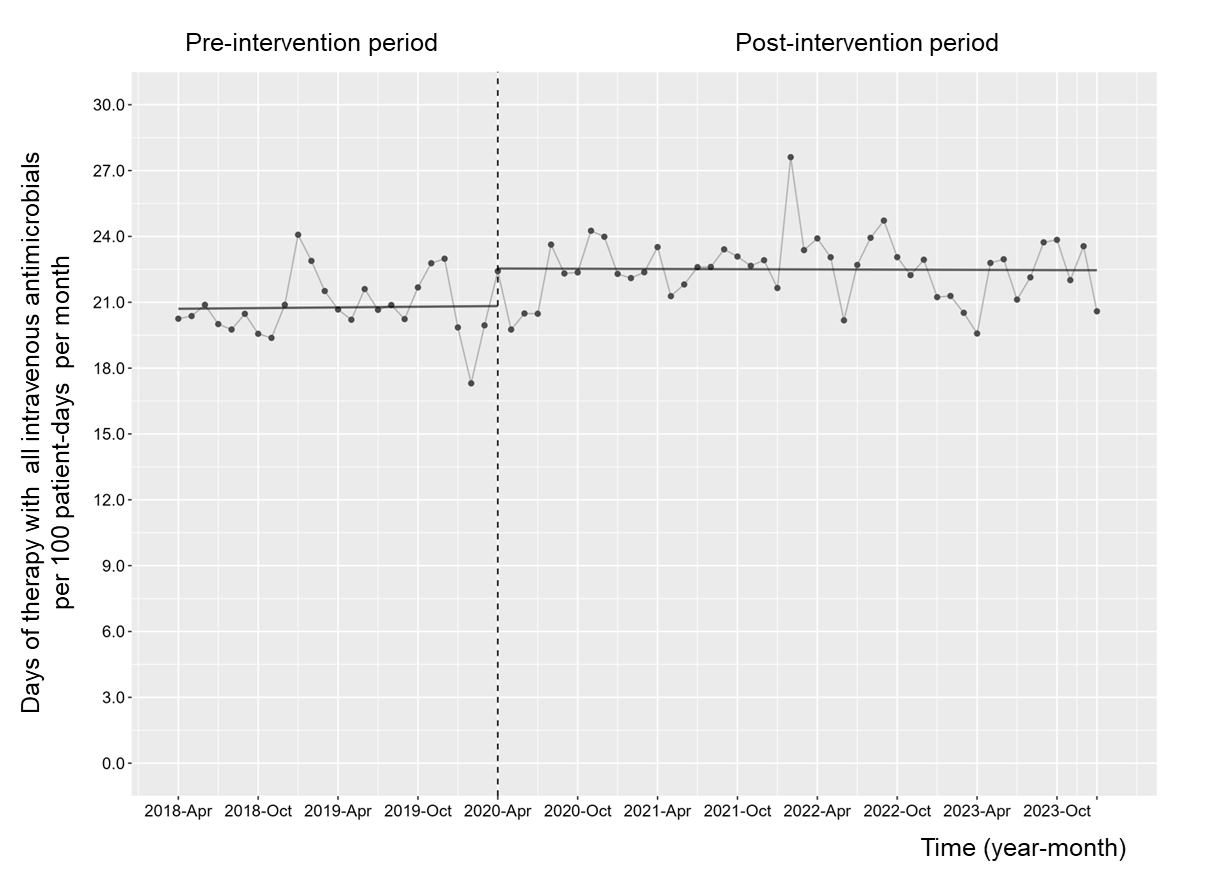
**

Each dot represents the days of therapy with all intravenous antimicrobials per 100 patient-days for each month. The slope is based on the linear regression analysis across two phases. The explanation of each phase is as follows: **Pre-intervention period** (antimicrobial notification by the infection control team from April 1, 2018, to March 31, 2020); **Post-intervention period** (establishing an infectious disease consultation service and implementation of the Antimicrobial Stewardship Program from April 1, 2020, to January 31, 2024). No significant change was observed in the level of the monthly DOT for the all-intravenous antimicrobials (coefficient: 1.71; 95% CI: -0.184 to 3.61, p = 0.08) or its trend (coefficient: -0.007; 95% CI: -0.13 to 1.11, p = 0.91).

**S7 Fig. The days of therapy with all oral antimicrobials per 100 patient-days per month**

**
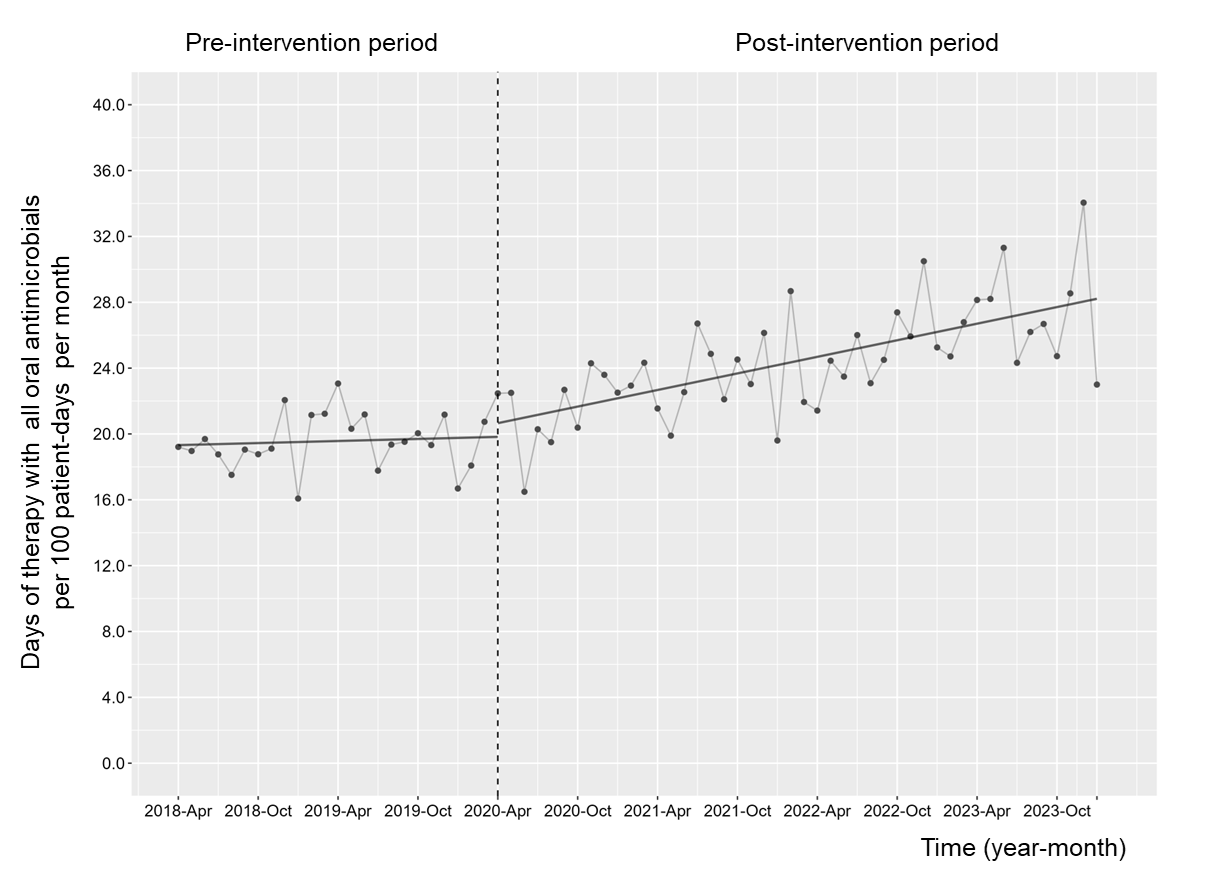
**

Each dot represents the days of therapy with all oral antimicrobials per 100 patient-days for each month. The slope is based on the linear regression analysis across two phases. The explanation of each phase is as follows: Pre-intervention period (antimicrobial notification by the infection control team from April 1, 2018, to March 31, 2020); Post-intervention period (establishing an infectious disease consultation service and implementation of the Antimicrobial Stewardship Program from April 1, 2020, to January 31, 2024). The trend of monthly all oral antimicrobials significantly increased (coefficient: 0.15, 95% CI: 0.03 to 0.27, p = 0.02), although its level did not decrease (coefficient: 0.83, 95% CI: -1.09 to 2.76, p = 0.39).

**S8 Fig.** **The days of therapy with** **the total of both intravenous and oral antimicrobials per 100 patient-days per month**


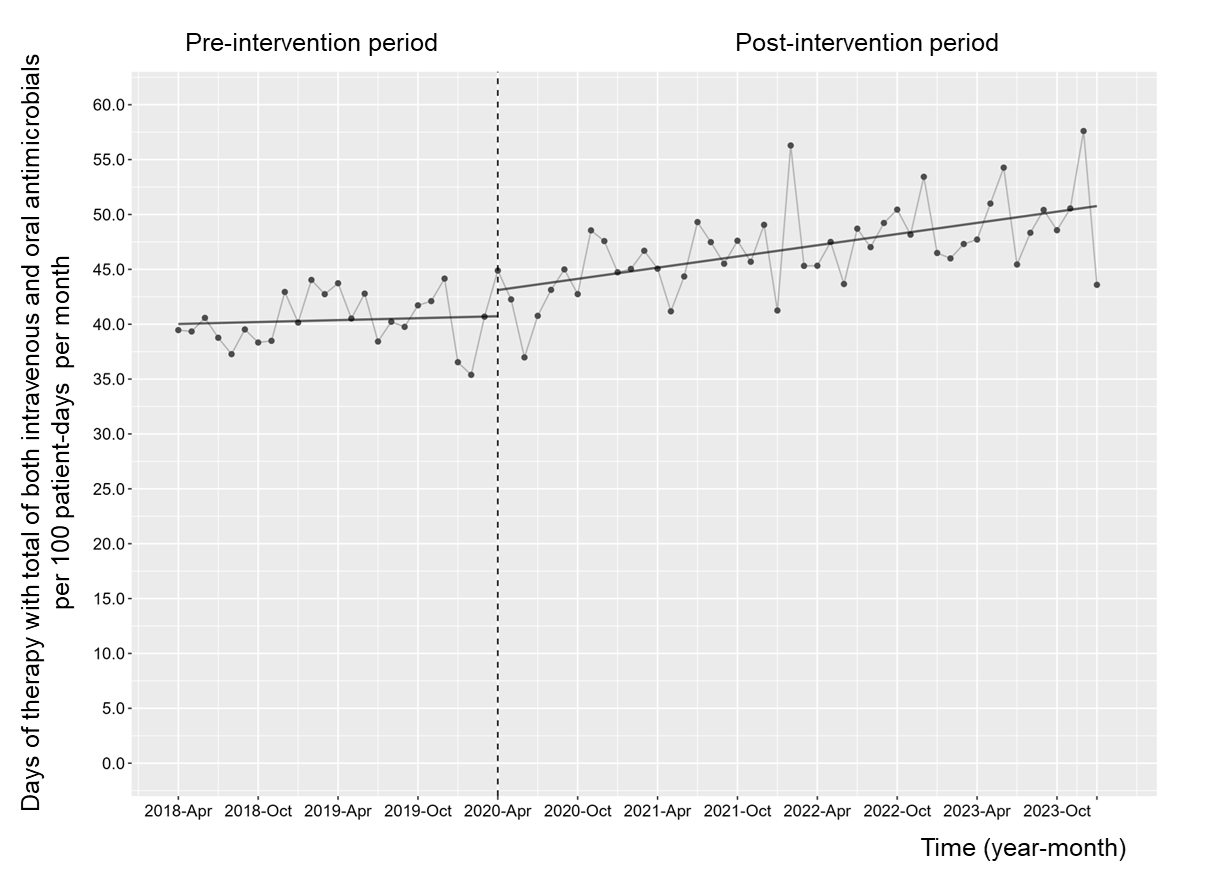


Each dot represents the days of therapy with all intravenous and oral antimicrobials per 100 patient-days for each month. The slope is based on the linear regression analysis across two phases. The explanation of each phase is as follows: Pre-intervention period (antimicrobial notification by the infection control team from April 1, 2018, to March 31, 2020); Post-intervention period (establishing an infectious disease consultation service and implementation of the Antimicrobial Stewardship Program from April 1, 2020, to January 31, 2024). No significant change was observed in the level of the monthly DOT for these antimicrobials (coefficient: 2.39; 95% CI: -0.34 to 5.12, p = 0.09) or its trend (coefficient: 0.14; 95% CI: -0.03 to 0.31, p = 0.10).

**S9 Fig. The incidence of CRE per 1,000 patient-days per month**


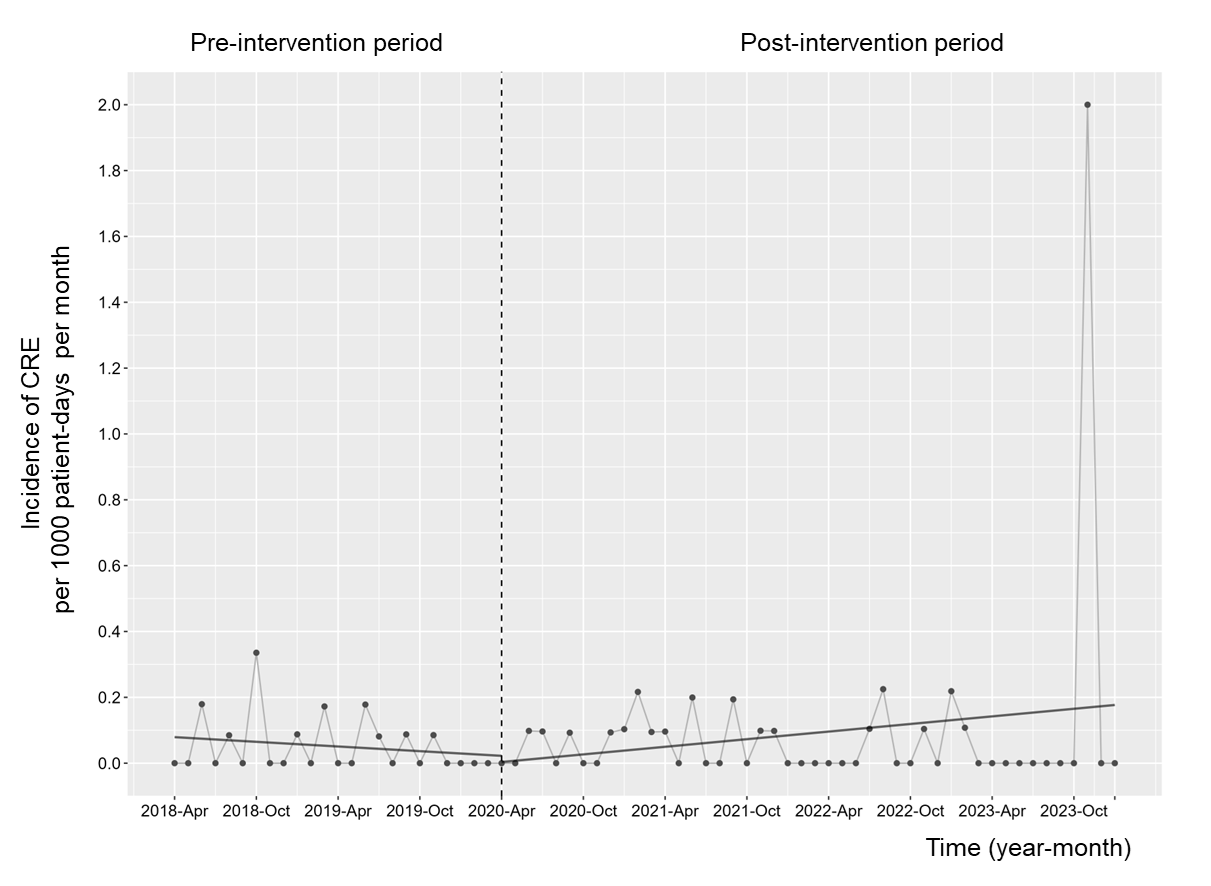


Each dot represents the incidence of carbapenem-resistant Enterobacterales (CRE) per 1,000 patient-days for each month. The slope is based on the linear regression analysis across two phases. The explanation of each phase is as follows: **Pre-intervention period** (antimicrobial notification by the infection control team from April 1, 2018, to March 31, 2020); **Post-intervention period** (establishing an infectious disease consultation service and implementation of the Antimicrobial Stewardship Program from April 1, 2020, to January 31, 2024). No significant change was observed in the level of the monthly incidence of CRE (coefficient: -0.02; 95% CI: -0.25 to 0.21, p = 0.87) or its trend (coefficient: 0.01; 95% CI: -0.01 to 0.02, p = 0.38).

**S10 Fig. The incidence of MRPA per 1,000 patient-days per month**


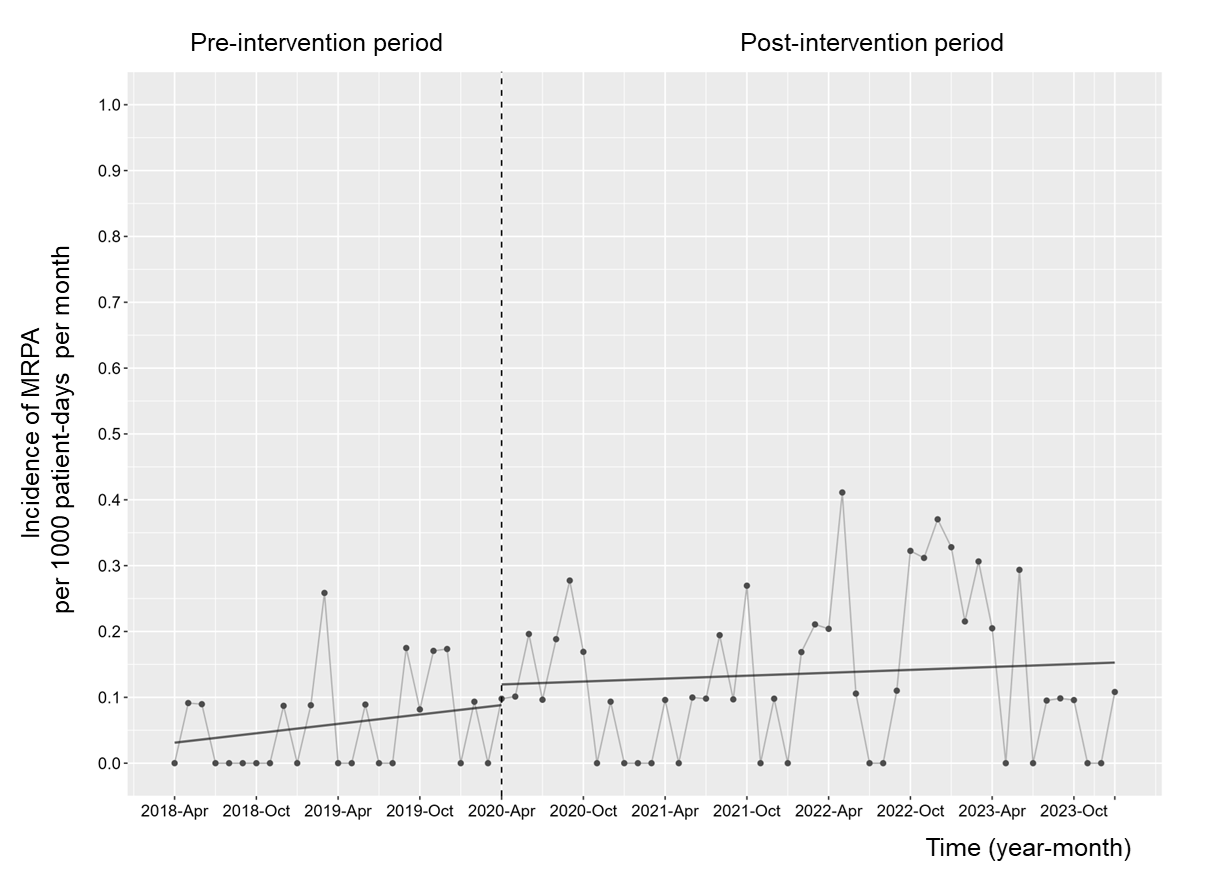
Each dot represents the incidence of multidrug-resistant *Pseudomonas aeruginosa* (MRPA) per 1,000 patient-days for each month. The slope is based on the linear regression analysis across two phases. The explanation of each phase is as follows: **Pre-intervention period** (antimicrobial notification by the infection control team from April 1, 2018, to March 31, 2020); **Post-intervention period** (establishing an infectious disease consultation service and implementation of the Antimicrobial Stewardship Program from April 1, 2020, to January 31, 2024). No significant change was observed in the level of the incidence of MRPA (coefficient: -0.02; 95% CI: -0.25 to 0.21, p = 0.87) or its trend (coefficient: 0.01; 95% CI: -0.01 to 0.02, p = 0.38).

**S11 Fig. The incidence of MRSA per 1,000 patient-days per month**


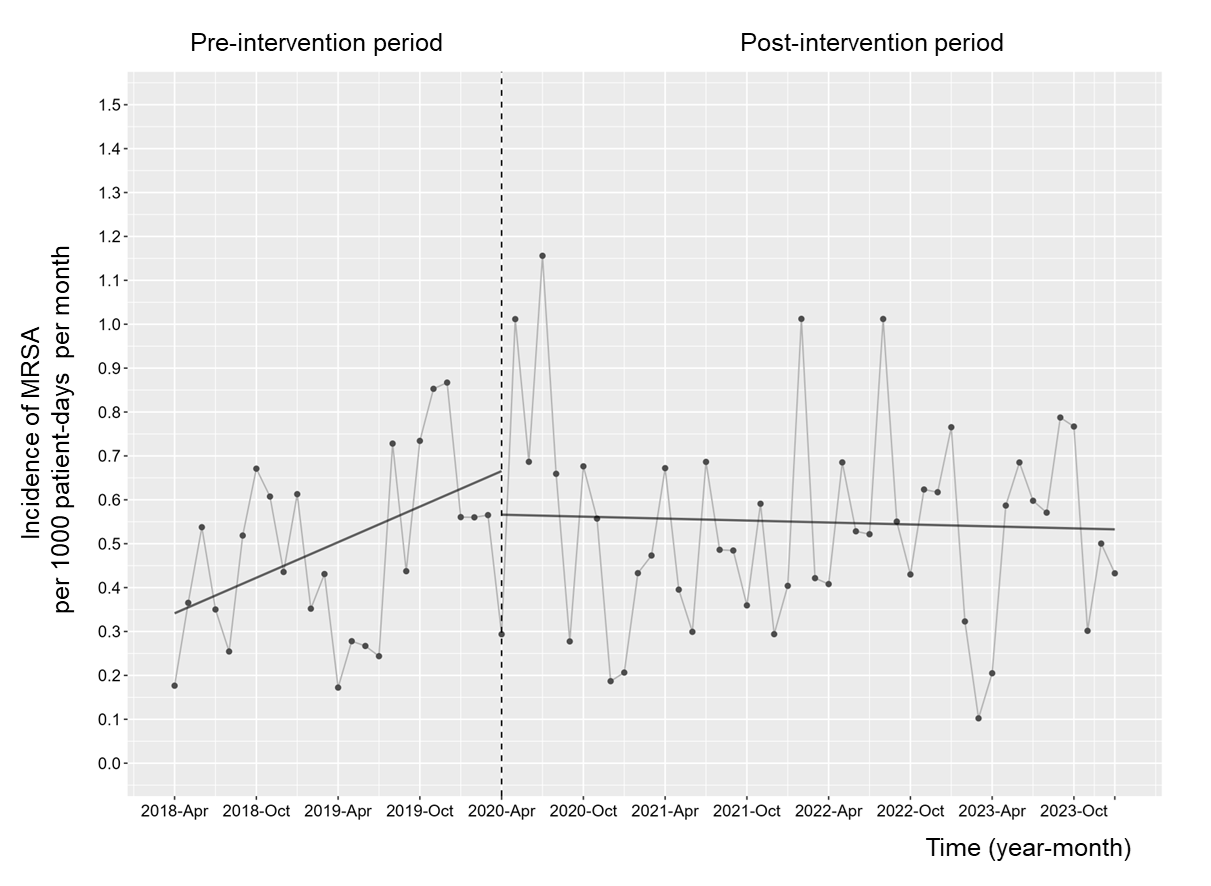
Each dot represents the incidence of methicillin-resistant *Staphylococcus aureus* (MRSA) per 1,000 patient days for each month. The slope is based on the linear regression analysis across two phases. The explanation of each phase is as follows: **Pre-intervention period** (antimicrobial notification by the infection control team from April 1, 2018, to March 31, 2020); **Post-intervention period** (establishing an infectious disease consultation service and implementation of the Antimicrobial Stewardship Program from April 1, 2020, to January 31, 2024). No significant change was observed in the level of the monthly incidence of MRSA (coefficient: 0.06; 95% CI: -0.09 to 0.20, p = 0.42) or its trend (coefficient: -0.01; 95% CI: -0.01 to 0.004, p = 0.28).

**S12 Fig. The incidence of ESBL-producing Enterobacterales per 1,000 patient-days per month**


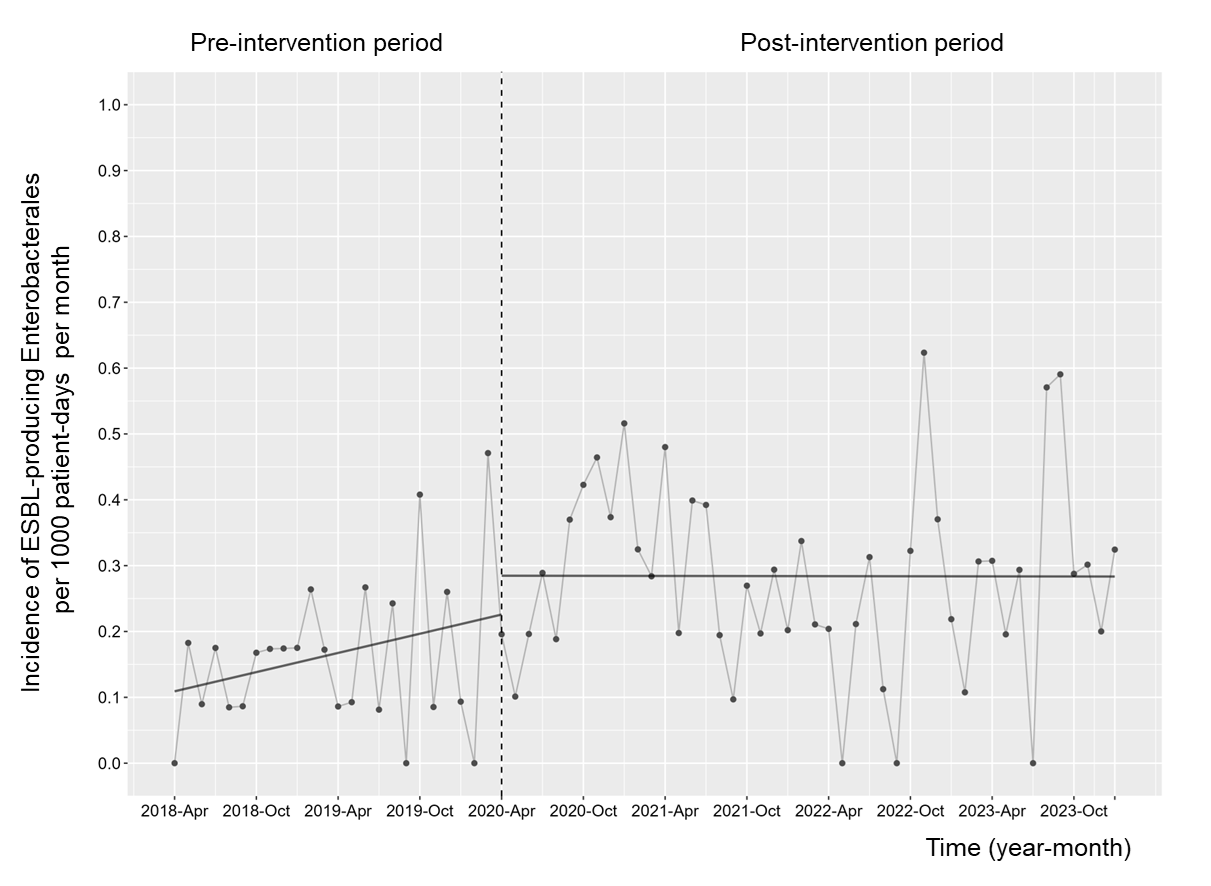


Each dot represents the incidence of extended-spectrum beta-lactamase (ESBL)-producing Enterobacterales per 1,000 patient-days for each month. The slope is based on the linear regression analysis across two phases. The explanation of each phase is as follows: **Pre-intervention period** (antimicrobial notification by the infection control team from April 1, 2018, to March 31, 2020); **Post-intervention period** (establishing an infectious disease consultation service and implementation of the Antimicrobial Stewardship Program from April 1, 2020, to January 31, 2024). There was no significant change in the level of the monthly incidence of ESBL-producing Enterobacterales (coefficient: -0.10; 95% CI: -0.36 to 0.16, p = 0.45) or its trend (coefficient: -0.01; 95% CI: -0.03 to 0.002, p = 0.09).

**S13 Fig. The incidence of CDI per 1,000 patient-days per month**


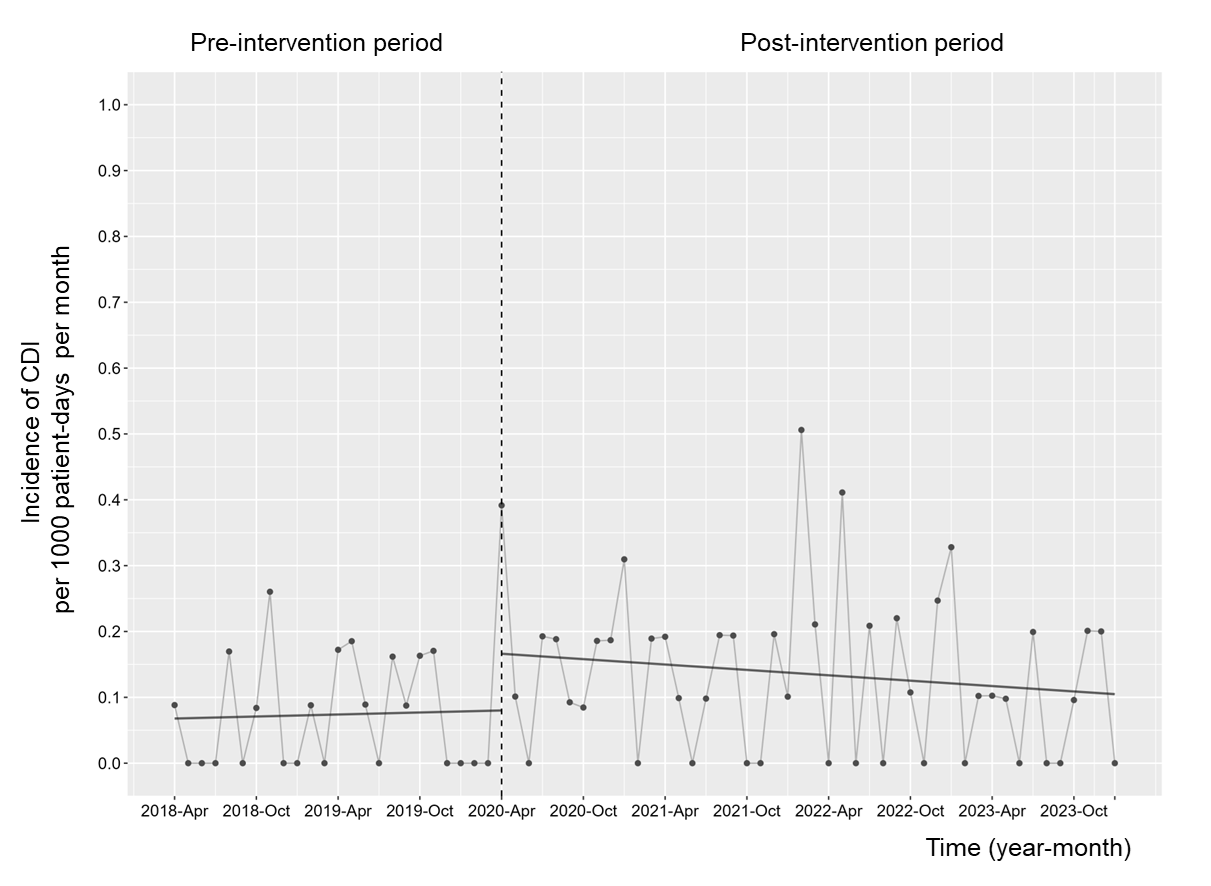


Each dot represents the incidence of *Clostridioides difficile* infection (CDI) per 1,000 patient-days for each month. The slope is based on the linear regression analysis across two phases. The explanation of each phase is as follows: **Pre-intervention period** (antimicrobial notification by the infection control team from April 1, 2018, to March 31, 2020); **Post-intervention period** (establishing an infectious disease consultation service and implementation of the Antimicrobial Stewardship Program from April 1, 2020, to January 31, 2024). There was no significant change in the level of the monthly incidence of CDI (coefficient: 0.09; 95% CI: -0.01 to 0.18, p = 0.08) or its trend (coefficient: -0.002; 95% CI: -0.008 to 0.004, p = 0.53).

**S14 Fig. The incidence of candidemia per 1,000 patient-days per month**


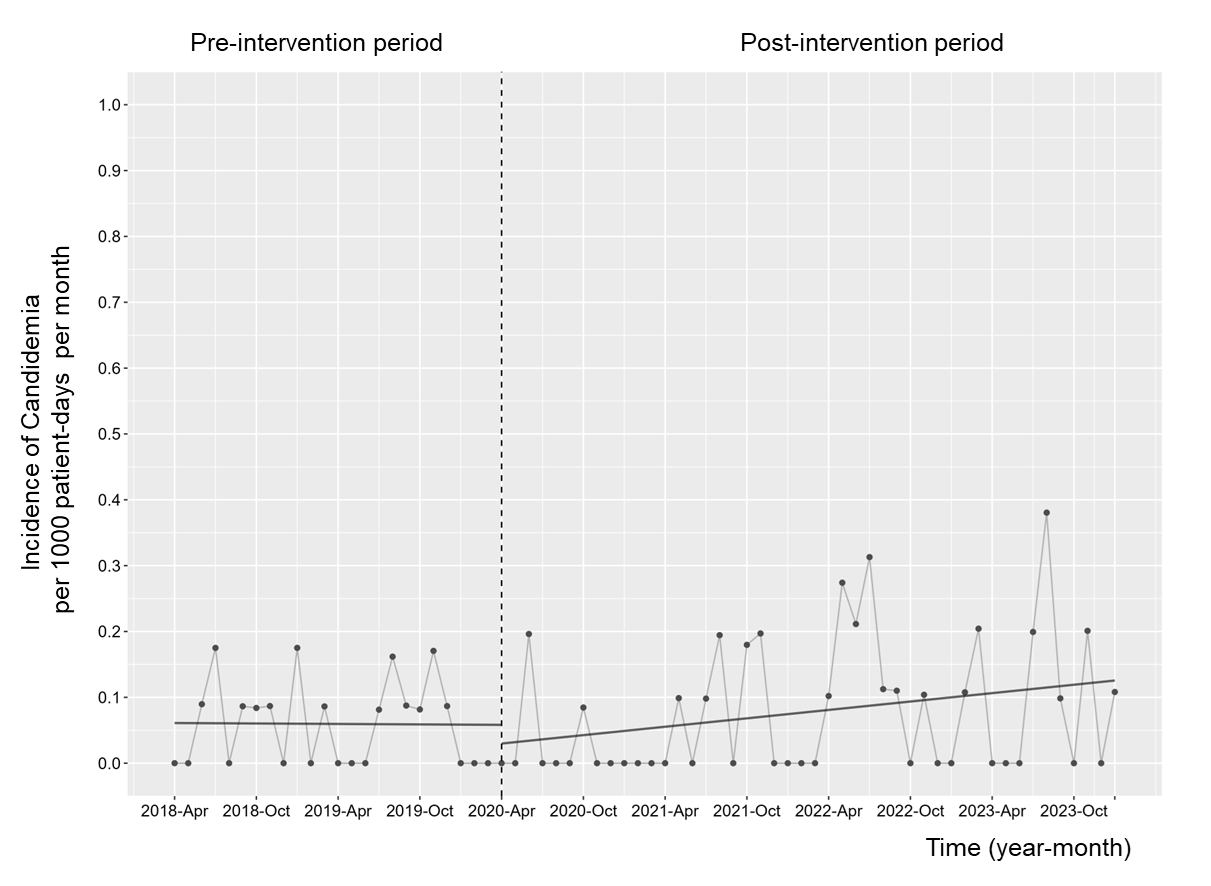


Each dot represents the incidence of candidemia per 1,000 patient-days for each month. The slope is based on the linear regression analysis across two phases. The explanation of each phase is as follows: **Pre-intervention period** (antimicrobial notification by the infection control team, April 1, 2018, to March 31, 2020); **Post-intervention period** (establishing an infectious disease consultation service and implementing the Antimicrobial Stewardship Program, April 1, 2020, to January 31, 2024). No significant change was found in the level of the monthly incidence of candidemia (coefficient: -0.03; 95% CI: -0.13 to 0.08, p = 0.58) or its trend (coefficient: 0.002; 95% CI: -0.004 to 0.009, p = 0.49).

**S15 Fig. The number of culture specimens per 1,000 patients per month**


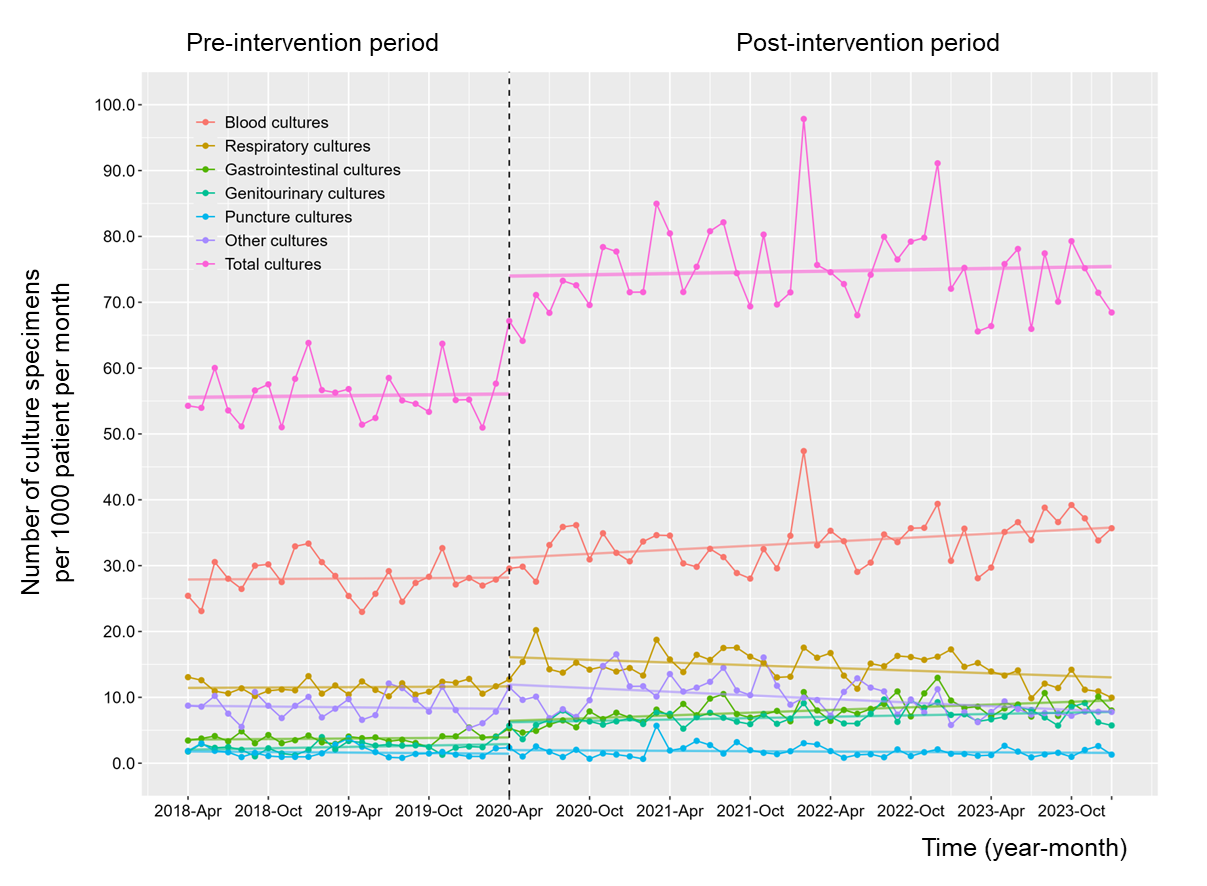


Each dot signifies the number of culture specimens per 1,000 patients each month, and the slope is calculated based on linear regression in two phases. The explanation of each phase is as follows: **Pre-intervention period** (antimicrobial notification by the infection control team from April 1, 2018, to March 31, 2020); **Post-intervention period** (establishing an infectious disease consultation service and implementation of the Antimicrobial Stewardship Program from April 1, 2020, to January 31, 2024). The analysis yielded the following results:

1) Total cultures: The levels significantly increased (coefficient for levels: 17.90, 95% CI: 11.6 to 24.2, p < 0.001), although its trend did not change (coefficient for trend: 0.01, 95% CI: -0.38 to 0.4, p = 0.96)

2) Blood cultures: Neither the levels nor trend exhibited a significant change (coefficient for levels: 3.00; 95% CI: -1.02 to 7.02, p = 0.14; coefficient for trend: 0.09; 95% CI: -0.16 to 0.34, p = 0.48)

3) Respiratory cultures: The levels significantly increased (coefficient for levels: 4.44, 95% CI: 2.26 to 6.62, p < 0.001), although its trend did not change (coefficient for trend: -0.08, 95% CI: -0.22 to 0.06, p = 0.26)

4) Gastrointestinal cultures: The levels significantly increased (coefficient for levels: 2.52, 95% CI: 1.15 to 3.89, p < 0.001), although its trend did not change (coefficient for trend: -‍0.06, 95% CI: -0.03 to 0.14, p = 0.20)

5) Genitourinary cultures: The levels significantly increased (coefficient for levels: 3.31, 95% CI: 2.18 to 4.45, p < 0.001), although its trend did not change (coefficient for trend: -0.0005, 95% CI: -0.071 to 0.07, p = 0.99)

6) Puncture fluid cultures: Neither the levels nor trend displayed a significant change (coefficient for levels: 0.55; 95% CI: -0.45 to 1.55, p = 0.28; coefficient for trend: 0.006; 95% CI: -0.06 to 0.07, p = 0.88)

7) Other cultures: The levels significantly increased (coefficient for levels: 3.70, 95% CI: 0.87 to 6.53, p = 0.01), although its trend did not change (coefficient for trend: -0.07, 95% CI: -0.25 to 0.11, p = 0.43)

**S16 Fig. The two-set rate of blood cultures per month**


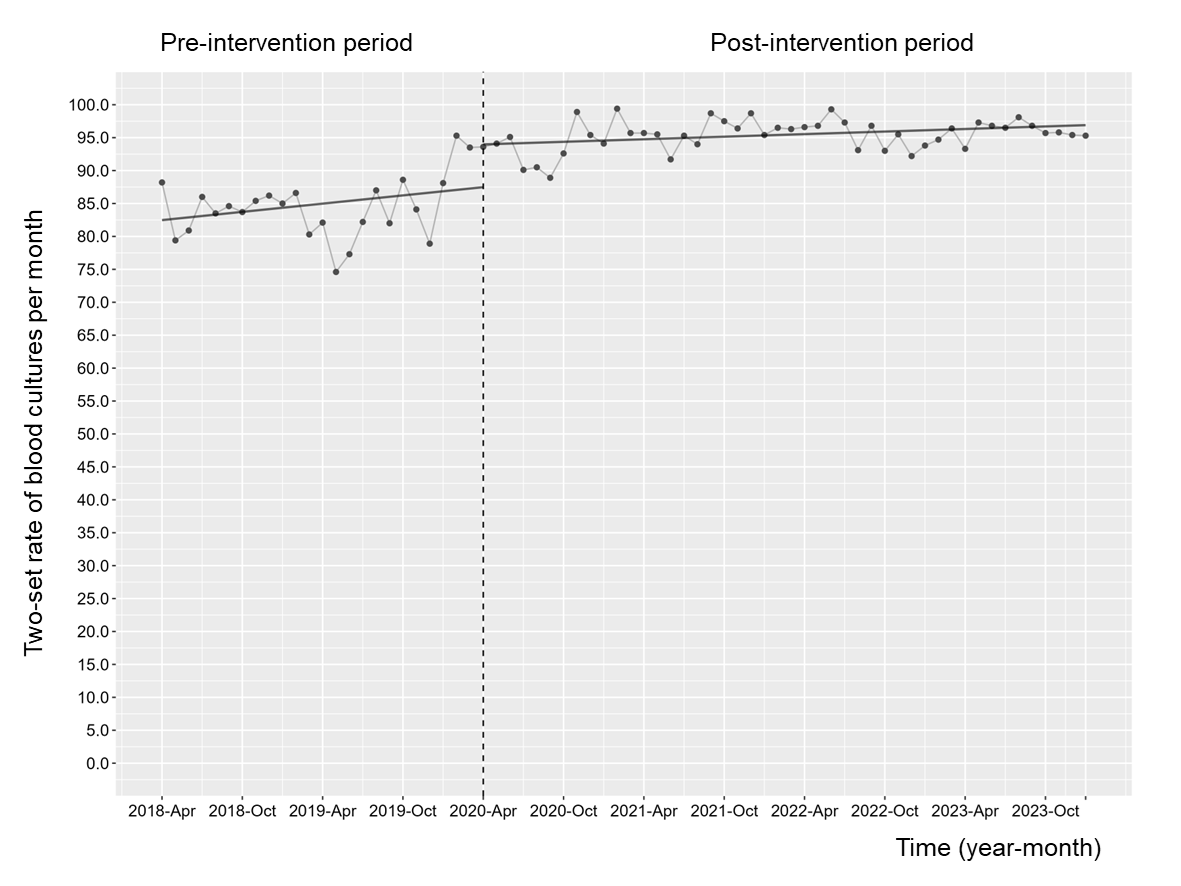


Each dot in the graph signifies the monthly two-set rate of blood cultures, with the slope calculated based on linear regression for the two phases. The explanation of each phase is as follows: **Pre-intervention period** (antimicrobial notification by the infection control team, April 1, 2018, to March 31, 2020); **Post-intervention period** (establishing an infectious disease consultation service and implementing the Antimicrobial Stewardship Program, April 1, 2020, to January 31, 2024). The trend in the two-set rate of monthly blood cultures did not increase after the intervention (coefficient -0.14; 95% CI: -0.42 to 0.14, p = 0.31), but the level did change significantly (coefficient 6.5; 95% CI: 2.16 to 10.8, p = 0.004).

**S17 Fig. The positive blood culture rate**


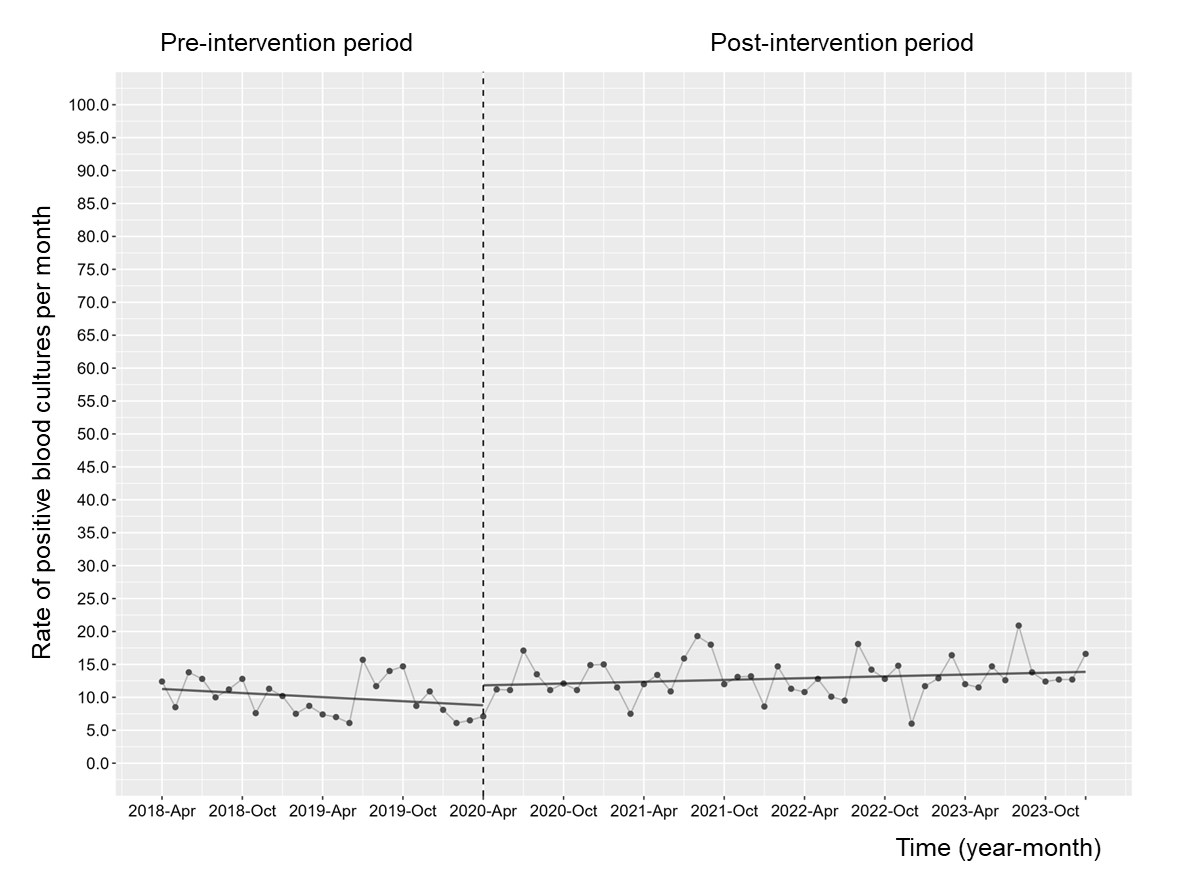


Each dot in the graph represents the monthly positive blood culture rate, with the slope calculated based on linear regression for the two phases. The explanation of each phase is as follows: **Pre-intervention period** (antimicrobial notification by the infection control team, April 1, 2018, to March 31, 2020); **Post-intervention period** (establishing an infectious disease consultation service and implementing the Antimicrobial Stewardship Program, April 1, 2020, to January 31, 2024). No significant change was observed in the level of the monthly positive blood cultures rate (coefficient: 3.03; 95% CI: -0.40 to 6.46, p = 0.08) or its trend (coefficient: 0.15; 95% CI: -0.07 to 0.36, p = 0.17).

**S18 Fig. All-cause 30-day mortality rate of patients with blood culture-positive episodes**


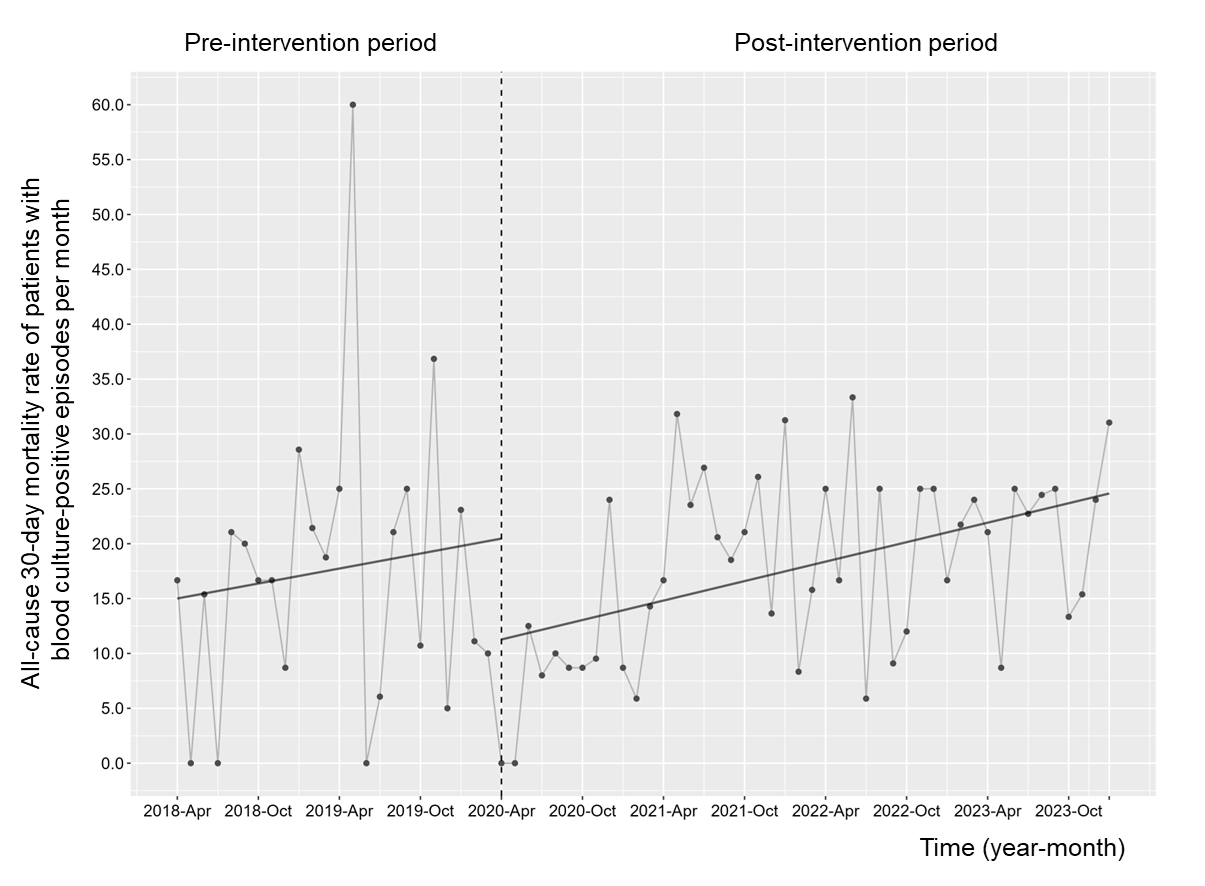


Each dot in the graph represents the monthly all-cause 30-day mortality rate of patients with blood culture-positive episodes, with the slope calculated based on linear regression for the two phases. The explanation of each phase is as follows: **Pre-intervention period** (antimicrobial notification by the infection control team, April 1, 2018, to March 31, 2020); **Post-intervention period** (establishing an infectious disease consultation service and implementing the Antimicrobial Stewardship Program, from April 1, 2020, to January 31, 2024). No significant change was found in the level of the monthly all-cause 30-day mortality rate of patients with blood culture-positive episodes (coefficient: -9.20; 95% CI: -18.4 to 0.01, p = 0.05) or its trend (coefficient: -0.07; 95% CI: -0.50 to 0.63, p = 0.81).

**S1 Table. Average monthly purchase cost per patient day for carbapenems and all intravenous antimicrobials from April 1, 2018, to January 31, 2024**

| Study period | Actual cost of carbapenems, USD | Adjusted cost of carbapenems, USD | Actual cost of all intravenous antimicrobials, USD | Adjusted cost of intravenous antimicrobials, USD |
| --- | --- | --- | --- | --- |
| April 1, 2018, to March 31, 2019 | 0.32 | 0.48 | 2.08 | 3.14 |
| April 1, 2019, to March 31, 2020 | 0.36 | 0.56 | 2.23 | 3.52 |
| April 1, 2020, to March 31, 2021 | 0.14 | 0.23 | 2.14 | 3.41 |
| April 1, 2021, to March 31, 2022 | 0.06 | 0.11 | 2.00 | 3.22 |
| April 1, 2022, to March 31, 2023 | 0.05 | 0.08 | 2.21 | 2.87 |
| April 1, 2023, to January 31, 2024 | 0.08 | 0.10 | 2.57 | 3.21 |

The actual cost includes the cost of switching to generic drugs and considers changes in drug prices.

The adjusted cost is calculated based on the drug price as of April 2024.

**S2 Table.** **Content and acceptance rate of feedback by the AST with regard to specific antimicrobial usage**

|  | Number of evaluations (n) | Suggestions (n) | Accepted (n) | Partially accepted (n) | Not accepted (n) | Acceptance rate^a^ (%) | Appropriate evaluations^b^ (%) |
| --- | --- | --- | --- | --- | --- | --- | --- |
| Early intervention period^c^ (n = 913) | 913 | 638 | 449 | 38 | 151 | 76.5 | 38.8 |
| Late intervention period^d^ (n = 1,686) | 1,686 | 1,119 | 865 | 62 | 202 | 82.8 | 52.7 |

^a^ Acceptance rate is the sum of accepted and partially accepted suggestions divided by the total number of suggestions.

^b^ Appropriate use is defined as A+B evaluation.

^c^ The early intervention period is defined as April 1, 2020, to March 31, 2021.

^d^ The late intervention period is defined as April 1, 2021, to January 31, 2024.

AST; Antimicrobial Stewardship Team.

**References**

1. Itoh N, Akazawa N, Kanawaku E, et al. Effects of infectious disease consultation and antimicrobial stewardship program at a Japanese Cancer Center: an interrupted time-series analysis. *PLoS One*. 2022;17:e0263095.

2. Centers for Disease Control and Prevention. Multidrug-resistant organism and Clostridioides difficile infection (MDRO/CDI) module. January 2020. Available at: https://www.cdc.gov/nhsn/PDFs/pscManual/12pscMDRO_CDADcurrent.pdf. Accessed July 19, 2024.

3. Clinical and Laboratory Standards Institute (CLSI). Performance standards for antimicrobial susceptibility testing 27th edn CLSI supplement M100-S27. Wayne: CLSI:PA2017.

4. Yonekawa S, Mizuno T, Nakano R, et al. Molecular and epidemiological characteristics of carbapenemase-producing Klebsiella pneumoniae clinical isolates in Japan. *mSphere*. 2020;5:e00490-20.

5. Akazawa T, Kusama Y, Fukuda H, et al. Eight-year experience of antimicrobial stewardship program and the trend of carbapenem use at a tertiary acute-care hospital in Japan-the impact of postprescription review and feedback. *Open Forum Infect Dis*. 2019;6:ofz389.
